# Modifying polyelectrolyte charge density and surface coverage for enhanced polyelectrolyte–surfactant lubrication of biomimetic interfaces

**DOI:** 10.1039/d6lf00165c

**Published:** 2026-07-31

**Authors:** Erik Weiand, Francisco Rodriguez-Ropero, Stefano Angioletti-Uberti, Daniele Dini, James P. Ewen

**Affiliations:** a Department of Mechanical Engineering, Imperial College London South Kensington Campus SW7 2AZ London UK erik.weiand19@imperial.ac.uk j.ewen@imperial.ac.uk; b Thomas Young Centre for the Theory and Simulation of Materials, Imperial College London South Kensington Campus SW7 2AZ London UK; c Corporate Functions Analytical and Data & Modeling Sciences, Mason Business Center, The Procter and Gamble Company Mason 45040 Ohio USA; d Department of Materials, Imperial College London South Kensington Campus SW7 2AZ London UK; e Department of Mechanical Engineering, University of Bath Claverton Down Bath BA2 7AY UK

## Abstract

Mixtures of polyelectrolytes and surfactants are useful components of many fluid formulations, including shampoos and conditioners. These mixtures form polyelectrolyte–surfactant complexes in solution that can adsorb on solid surfaces and significantly reduce friction. Experiments have shown that the charge density of polyelectrolytes affects their adsorption and conformation on the surface, which are expected to strongly influence their lubrication performance. Here, we use coarse-grained, non-equilibrium molecular dynamics simulations to study how different charge densities and surface coverages of a polyelectrolyte, cationic guar gum, in the presence of an anionic surfactant, sodium dodecyl sulfate, influence hair friction. We observe improved lubrication for polyelectrolytes with higher charge densities on biomimetic surfaces representative of both virgin and bleached hair. This is attributed to increased surface separation, which is facilitated by higher retention of interfacial surfactants and water. Higher polyelectrolyte surface coverages also result in lower friction through the same mechanism. At sufficiently high polyelectrolyte surface coverages, friction becomes insensitive to the level of chemical damage on the hair surface. Most polyelectrolytes have flat, train-like conformations, particularly at high charge density and low surface coverage. The number of tail and loop conformations increases at lower charge density and higher surface coverage, but this has a minor effect on friction compared to the increased film thickness. These simulations show considerable promise for virtually screening polyelectrolytes with optimal lubricity performance in liquid formulations.

## Abstract 2

Coarse-grained molecular dynamics show that mixtures of polyelectrolytes and surfactants are effective at reducing friction on hair surfaces, particularly with increasing polyelectrolyte coverage.

## Graphical abstract

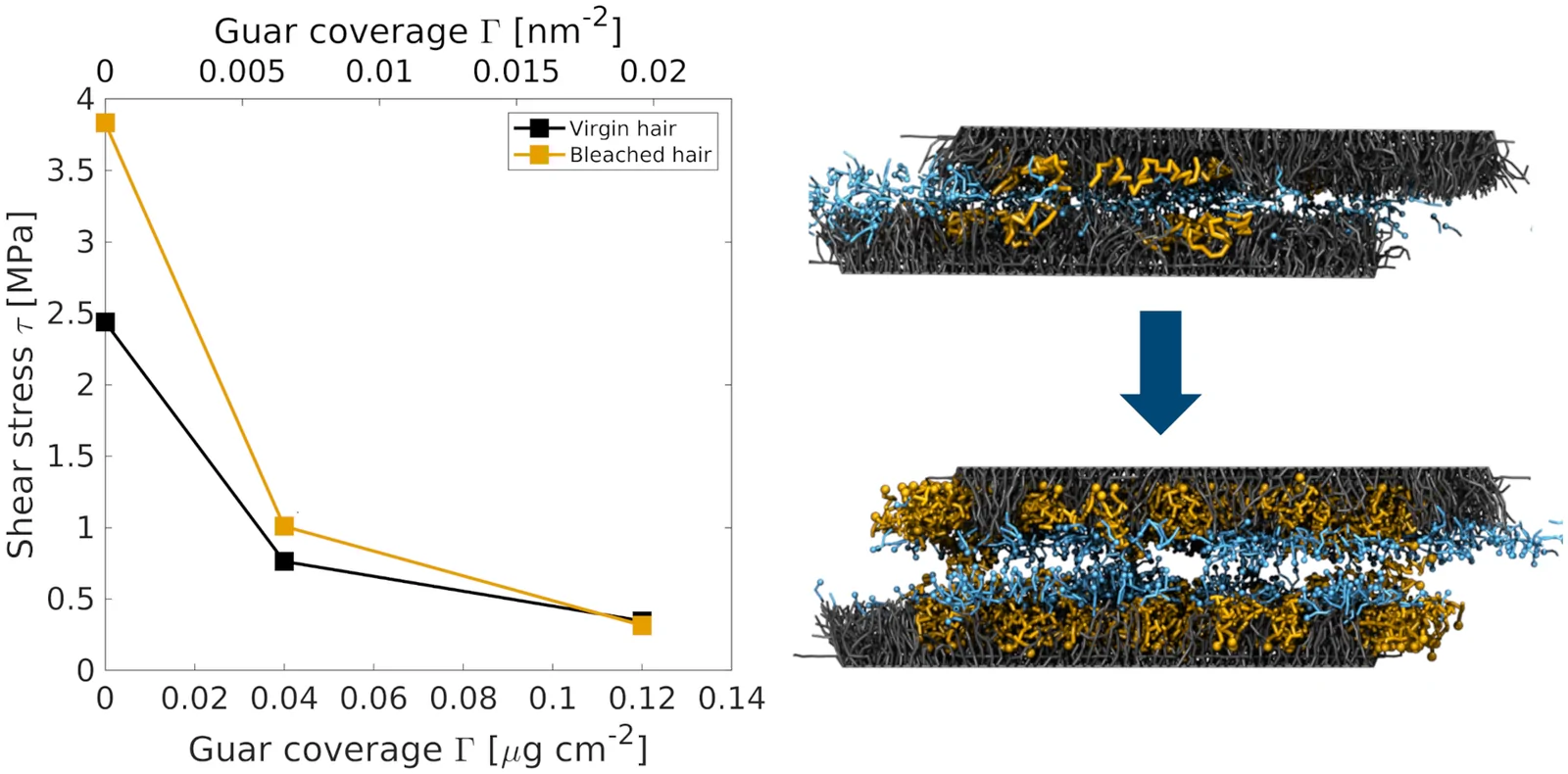

## Introduction

The development of personal care products with reduced impact on the environment is a key challenge for formulators in the cosmetic industry.^1^ This includes replacing fossil-derived synthetic ingredients with those that can be sourced from natural products.^2^ Charged surfactants and polyelectrolytes are key components of many home and personal care formulations,^3^ particularly shampoos and conditioners.^4,5^ They can be sourced from both fossil sources and natural or bio-sources.^2^ Cationic surfactants and polyelectrolytes are used mainly for hair conditioning, due to their strong adsorption on negatively charged patches of the hair.^6^ These anionic regions are more prevalent on hair that has been bleached or chemically damaged.^7,8^ Anionic surfactants are primarily used to modify viscosity and remove excess fats and dirt from the surface, thus fulfilling the role of a cleansing agent.^5^

Combining oppositely charged compounds can lead to the formation of aggregates or complexes. The so-called ‘dilution deposition’ mechanism enables the efficient surface adsorption of additives in formulations.^9,10^ After applying a shampoo or conditioner containing a single-phase solution, rinsing with water leads to a dilution of the oppositely-charged polyelectrolytes and surfactants contained in the formulation.^9,10^ This can induce a separation into two phases, which is driven by a release of counterions and the resultant increase in entropy.^11^ The dense phase or ‘coacervate’ contains high concentrations of the oppositely charged compounds and favorably adsorbs to the hair surface.^12,13^ The dilute (or supernatant) phase is typically easily rinsed off the hair surface. For a detailed description of the coacervation process, the ‘dilution deposition’ mechanism and corresponding phase diagrams, we refer the reader to other works.^10–12,14^ The investigation of coacervation and dilution deposition has been found to be challenging due to inherent non-equilibrium phenomena and macroscopic timescales required to reach true equilibrium conditions.^15^ In this study, we therefore use a simplified simulation protocol deliberately circumnavigating around the very long timescales required to reach equilibrium coacervates.

Previous experiments of adsorption of formulations containing oppositely charged polyelectrolyte–surfactant complexes reported film thicknesses up to several microns on hair-like surfaces.^16–21^ The structure of the adsorbed polyelectrolyte films depends on the polymer charge density, with higher values leading to more compact conformations.^22^ For polyelectrolyte–surfactant complexes, low charge density polymers generally have extended pearl-necklace structures, where surfactant micelles attach to oppositely charged binding sites along the polymer.^23^ At high polymer charge density, these structures tend to collapse.^23^

Reducing hair friction is important for a positive consumer perception of hair care products.^24^ The implications of adsorbed polymer–surfactant complexes on hair lubrication are not yet fully understood. Friction reductions due to the application of oppositely-charged polymers and surfactants have been reported experimentally for both real hair and biomimetic surfaces.^25,26^ Previous works hypothesized that effective lubrication could arise from the formation of loops^25^ or brush-like conformations^27,28^ by the adsorbed polyelectrolytes. Polymer brushes, particularly those that form loops, are known to effectively reduce friction in the boundary lubrication regime.^29^ It is also conceivable that these extremely viscous polyelectrolytes–surfactant complexes could reduce friction by separating the sliding hairs through hydrodynamic lift.^30,31^ However, the relationship is non-trivial, as outlined in sensory evaluation tests of stickiness and viscosity, and strongly depends on the polymer–surfactant concentrations and ratio.^14^

Molecular dynamics (MD) simulations are a promising tool for studying the lubrication of oppositely-charged polyelectrolyte–surfactant systems at hair–hair interfaces. Coarse-grained (CG) force fields enable long dynamic simulations of large macromolecules to capture important behavior or polyelectrolytes and even coacervation.^32,33^ Recently, coacervation has been simulated successfully with CG-MD^34–37^ using the MARTINI force field.^38,39^ MARTINI^38^ has also been used to study the adsorption and lubrication of polyelectrolyte–surfactant complexes with hair surfaces.^40,41^ In a previous study,^41^ we assessed the boundary lubrication performance between two biomimetic hair surfaces in the presence of thin layers of oppositely charged polyelectrolyte–surfactant complexes. We provided fundamental insights on how a naturally-sourced polyelectrolyte, cationic guar gum (CGG) with high charge density, can drastically reduce friction through synergistic effects with the anionic surfactant sodium dodecyl sulfate (SDS).^41^ We considered relatively low polyelectrolyte surface coverage,^41^ similar to those measured experimentally for biomimetic surfaces representative of virgin hair.^16^ The high charge density and low surface coverage lead to flat polyelectrolyte conformations without tails or loops,^22^ which have been hypothesized to be important for the effective lubrication of polyelectrolyte–surfactant complexes.^25^ Recent implicit solvent CG-MD deposition studies on flat biomimetic hair surfaces highlighted a strong dependency of the polymer architecture on the adsorption density and resistance to external shear flow.^42,43^ Experimentally, there is also evidence of swelling of polyelectrolyte layers on hydrophilic surfaces upon addition of anionic surfactants near or above the critical micelle concentration (cmc).^16^ In our previous study,^41^ negligible SDS-induced swelling of the polyelectrolyte layer was observed during the adsorption simulations, which could be due to the high CGG charge density employed.

In this work, we systematically assess different properties of CGG with respect to their nanoscale hair–hair lubrication performance in the presence of anionic surfactants (SDS) using a coarse-grained biomimetic hair surface model. The MARTINI 2 force field^38^ is used for the polyelectrolytes, surfactants, and hair surfaces. We first investigate the effect of the polyelectrolyte charge density on complexation with SDS, adsorption on hair, and lubrication performance. We also study the effect of increased polyelectrolyte surface coverage, which is representative of the levels observed experimentally on bleached.^44^ This systematic parameter variation is expected to enable further fine-tuning of the polysaccharide synthesis and selection processes for hair care products with excellent lubricity performance.

## Methods

### Polyelectrolyte parameters

**Fig. 1:**
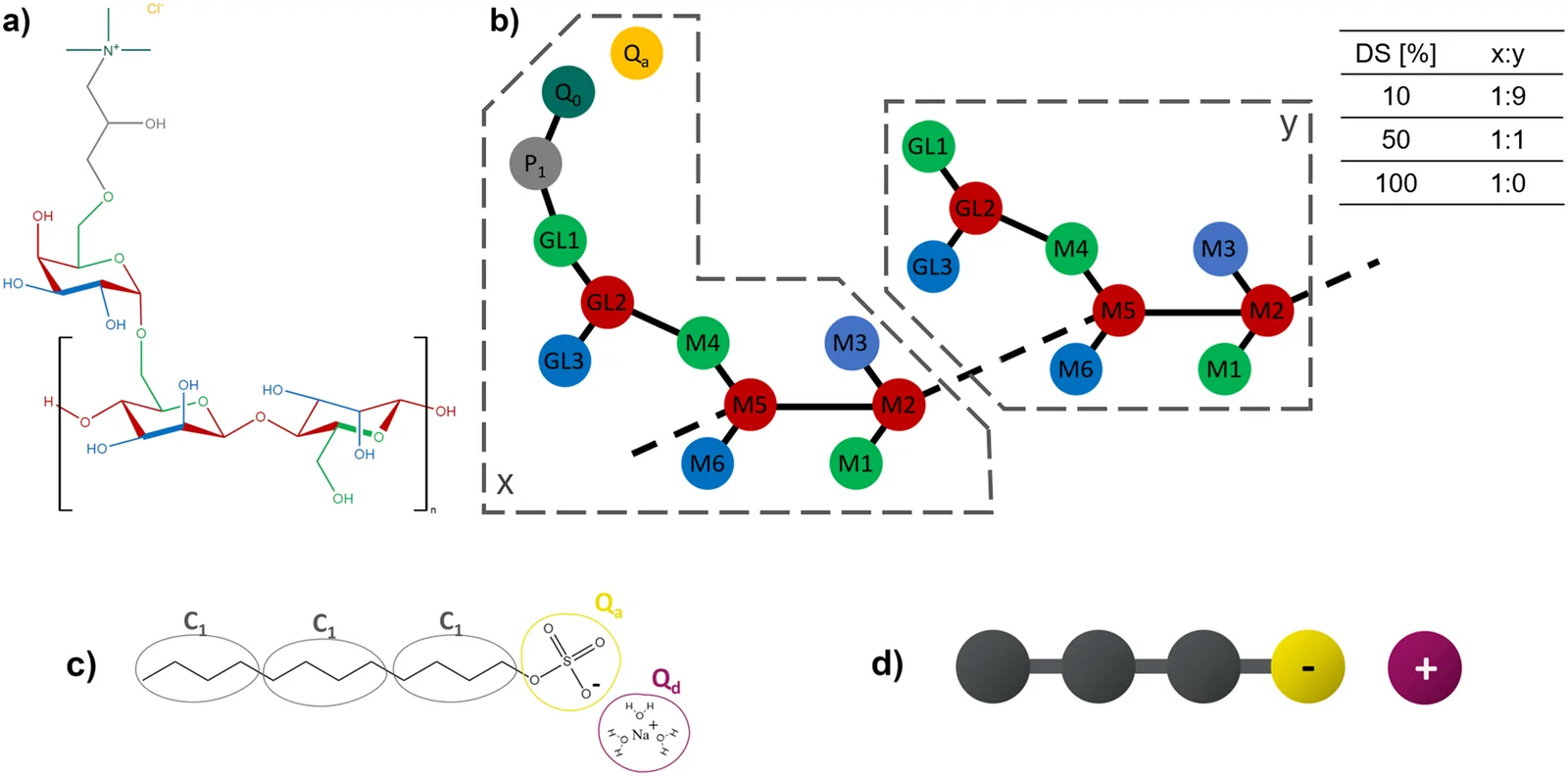
Molecules of interest for adsorption: (a and b) cationic guar gum, CGG and (c and d) sodium dodecyl sulfate (SDS). The degree of substitution (DS) for CGG is determined by the ration between *x* and *y* as shown in (b). Adapted from ref. 45 under a CC-BY 3.0 license and from ref. 41 under a CC-BY 4.0 license.

We follow a three-step protocol for simulating friction between two hair surfaces, as used in previous works:^41,45,46^ (1) adsorption of compounds, (2) squeeze-out, and (3) sliding non-equilibrium MD (NEMD) simulations. The squeeze-out step has been deemed critical to capturing lubrication across different normal loads.^41,45,46^ It consists of a compression simulation of a lubricant film between two hair surfaces in an explicit water bath. The surfaces are compressed at constant normal force until an equilibrium with respect to the film thickness and composition between the two surfaces is observed. An overview of the systems considered for our adsorption, squeeze-out and sliding MD simulations is shown in Table 1. The molecules considered in this work, namely CGG at a molecular weight of 36.9 kDa and SDS, are shown in Fig. 1. In our previous study,^41^ we considered CGG, guar hydroxypropyltrimonium chloride, with a degree of substitution (DS) of 100%, meaning one quaternary ammonium group added to every galactose side branch. In this work, we introduce systems with a DS of 10 and 50% (cases I–II) in addition to the baseline case (III). The lower values are closer to CGG degrees of substitution in the range of 4 to 30% as reported experimentally for CGG in the literature.^47,48^ In our previous study,^41^ we also considered a relatively low CGG surface coverage of *Γ* = 0.04 μg cm^−2^ that is representative of experimental ellipsometry measurements of the adsorption density of CGG on a hydrophobic surface,^16^ which is similar to virgin hair.^49^ Here, we also carry out simulations at a higher CGG surface coverages, *Γ* = 0.12 μg cm^−2^ (case IV). Similar adsorption densities were reported for CGG using quartz crystal balance with dissipation monitoring (QCM-D) and ellipsometry measurements on sulfonic acid-functionalised anionic surfaces,^44^ which are similar to bleached hair.^49^

**Table 1:** Overview of CG-MD simulation cases for the CGG architecture parameter study. The baseline case (III) as presented in ref. 41 is denoted in bold

Test case	*N*_mon_	DS	*Γ*_0,CGG_
I	50	10%	0.04 μg cm^−2^
II	50	50%	0.04 μg cm^−2^
**III**	**50**	**100%**	**0.04 μg cm** ^**−2**^
IV	50	50%	0.12 μg cm^−2^

For polymers where DS < 100%, we also carried out preliminary adsorption simulations with different charge functionalization patterns. This was motivated by previous simulations that showed differences in polyelectrolyte adsorption for uniform (blocky), regular, and randomly arranged surface charges.^50^ We tested uniform, regular, and randomly arrangements of the charged groups along the polyelectrolyte backbone. In real CGG, the charge pattern will depend on the functionalization process.^51,52^ Our preliminary adsorption simulations on both virgin and bleached model hair surfaces did not indicate any strong differences in the structure of the adsorbed chains. Therefore, all subsequent simulations were carried out using CGG with regular charge patterns, *i.e.* alternating charged/uncharged repeat units for DS = 50% and one charged in every ten for DS = 10%.

The polyelectrolyte topology may also affect its adsorption and friction behaviour. Previous coarse-grained MD simulations observed only minor differences between the adsorption behavior of linear and branched, comb-like cationic polyelectrolytes.^43^ Moreover, the naturally-sourced polyelectrolytes of interest in this study are usually linear.^43^ Therefore, only linear polymers were considered in our simulations.

### Simulation protocol

We use biomimetic models of the surfaces of both virgin and medium bleached hair we developed and validated previously.^53^ Virgin hair contains 75% fatty acid groups and 25% sulfonate groups, while bleached hair contains 15% fatty acid groups and 85% sulfonate groups, as established by numerical and experimental contact angle data.^53^ These represent the healthy 18-methyleicosanoic acid (18-MEA) layer bound to underlying cysteine proteins, which is oxidised to anionic cysteic acid upon bleaching.^7,8,49^ The degree of sulfonate group coverage was motivated by previous TOF-SIMS experiments.^54^

We adopted a sequential adsorption procedure for CGG and SDS on the hair surfaces, as outlined in our previous study.^41^ It is important to note that the structure of the adsorbed polyelectrolyte–surfactant complexes on the surface could depend on how they are deposited. Dedinaite *et al.*^15^ showed that the transition from quasi-equilibrium polyelectrolyte–surfactant adsorption to true equilibrium can only be reached on timescales beyond those accessible to most experimental studies (days). Even using coarse-grained force fields and enhanced sampling methods, these timescales are well beyond those that can be accessed with MD simulations.^55^ Therefore, we cannot claim that the adsorbed polyelectrolyte–surfactant complex structures used in this work are at true equilibrium. This means that our simulation approach does not directly resemble the realistic deposition process where deposition of large pre-assembled aggregates from solution and possibly the formation of surfactant layers prior to polymer deposition could occur. Recent adsorption experiments of the cationic polyelectrolyte chitosan and SDS to biomimetic surfaces representative of partially damaged hair^19^ directly compared the structure of adsorbed complexes for sequential adsorption and adsorption from a pre-mixed solution. The structure and film thickness for the two approaches were in close agreement.^19^ This supports the idea that the sequential approach chosen in our simulations can provide relevant insights into the physicochemical mechanisms of hair care formulations. It would also be valuable to systematically test the adsorption of pre-aggregated complexes in simulations, such as those performed by Coscia *et al.* at constant composition and contact pressure.^40^ Implicit solvent force fields, increased coarse graining, and enhanced sampling techniques could provide the required speed-up compared to the simulations reported in the current study.

After initial adsorption of CGG chains, pre-assembled SDS micelles were added into the bulk solution at a concentration of *c* = 664 mM. A micelle aggregation number of *N* = 45 was used for the pre-assembled micelles, which is similar to previous CG-MD studies.^46,56^ The SDS adsorption simulations were then run for 50 ns. The size of the systems for the adsorption and squeeze-out simulations was generally 40 × 21 × 32 nm.^41^ For systems with increased CGG chain length or increased adsorption densities, the *z*-dimension of the simulation box size was increased (up to 38 nm) for the adsorption and squeeze-out simulations.

For the squeeze-out stage, the depletion of CGG and SDS from the contact was studied at a constant contact pressure of *σ* = 10 MPa. This is within the range expected for hair–hair contacts at the loads anticipated during hair manipulation.^46^ When the surface separation reached an equilibrium value after approximately 50–100 ns, the contact composition and thickness were extracted. Partial squeeze-out of CGG is expected for cases with low polymer charge density or high initial adsorption density. The number of CGG chains for the NEMD simulations was rounded to the nearest even number obtained from squeeze-out to ensure the same number of polymers on each surface.

For the NEMD simulations, excess CGG chains were removed based on their distance from the substrate. CGG deletion was based on the topology obtained at the end of the CGG adsorption stage to ensure that the system retains its periodicity in *x*- and *y*-directions. Prior to the NEMD simulations, SDS and water were then re-introduced in a slab at the center of the CGG-adsorbed contact using the updated composition from the squeeze-out simulations. This is a limitation of our squeeze-out routine and could lead to differences adsorption structures at the NEMD stage compared to the end of the squeeze-out stage. Semi-periodic (*x* & *y*) NEMD simulations are run at a fixed sliding velocity of *v*_s_ = 0.1 ms^−1^. In this study, we only consider a constant sliding velocity, which is deemed representative of physiologically relevant hair manipulations.^46^ In our previous study of polymer–surfactant complexes, we observed a power-law dependence of the shear stress on the sliding velocity,^41^ which has also been reported from experiments of comparable systems.^28,57^ Similar power-law scaling is expected for the systems studied here, but this is not the key focus of this work.

### Simulation details

The simulation setup in this work closely follows our previous work on the boundary lubrication performance of CGG–SDS complexes on hair.^41^ All MD simulations are performed using the open-source code LAMMPS.^58^ Velocity-Verlet integration is used with a timestep of 5 fs. Moltemplate^59^ and PackMol^60^ are used to apply the force field parameters and assemble the molecular systems. The system temperature is maintained at 300 K using a Langevin thermostat^61^ applied only in the *y*-direction and to the base bead of the surface-grafted molecules (18-MEA and cysteic acid).

We used MARTINI 2.0 (ref. 38 and 62) parameters for the hair surfaces^53^ CGG, and SDS, with the mappings shown in Fig. 1. The polarizable MARTINI water model^63^ is used throughout. The bonds between the central and satellite beads of water molecules are constrained to *r* = 1.4 nm using the SHAKE algorithm.^64^ Lennard-Jones (LJ) interactions between coarse-grained beads are in accordance with ref. 62 and 63 and are smoothly shifted to zero between 0.9 nm and 1.2 nm. Direct coulombic interactions between beads are considered below a cutoff radius of 0.9 nm. At larger distances, a slab adaption^65^ of the particle–particle particle–mesh (PPPM) method^66^ is used. System charge neutrality is ensured by introducing randomly distributed sodium or chloride counterions. Bonded interactions are considered by means of harmonic bond and angle potentials as provided in detail in our previous work.^41^

## Results and discussion

### Effect of the polymer charge density

The polyelectrolyte charge density is expected to influence both surface adsorption^16^ and friction.^40^ We therefore performed adsorption, squeeze-out and friction simulations with CGG at reduced charge densities corresponding to a degree of substitution (DS) of 10% and 50% (cases I and II). These new simulations are compared to our baseline (case III), as presented in full in our previous study.^41^

### Adsorption on single surfaces

**Fig. 2:**
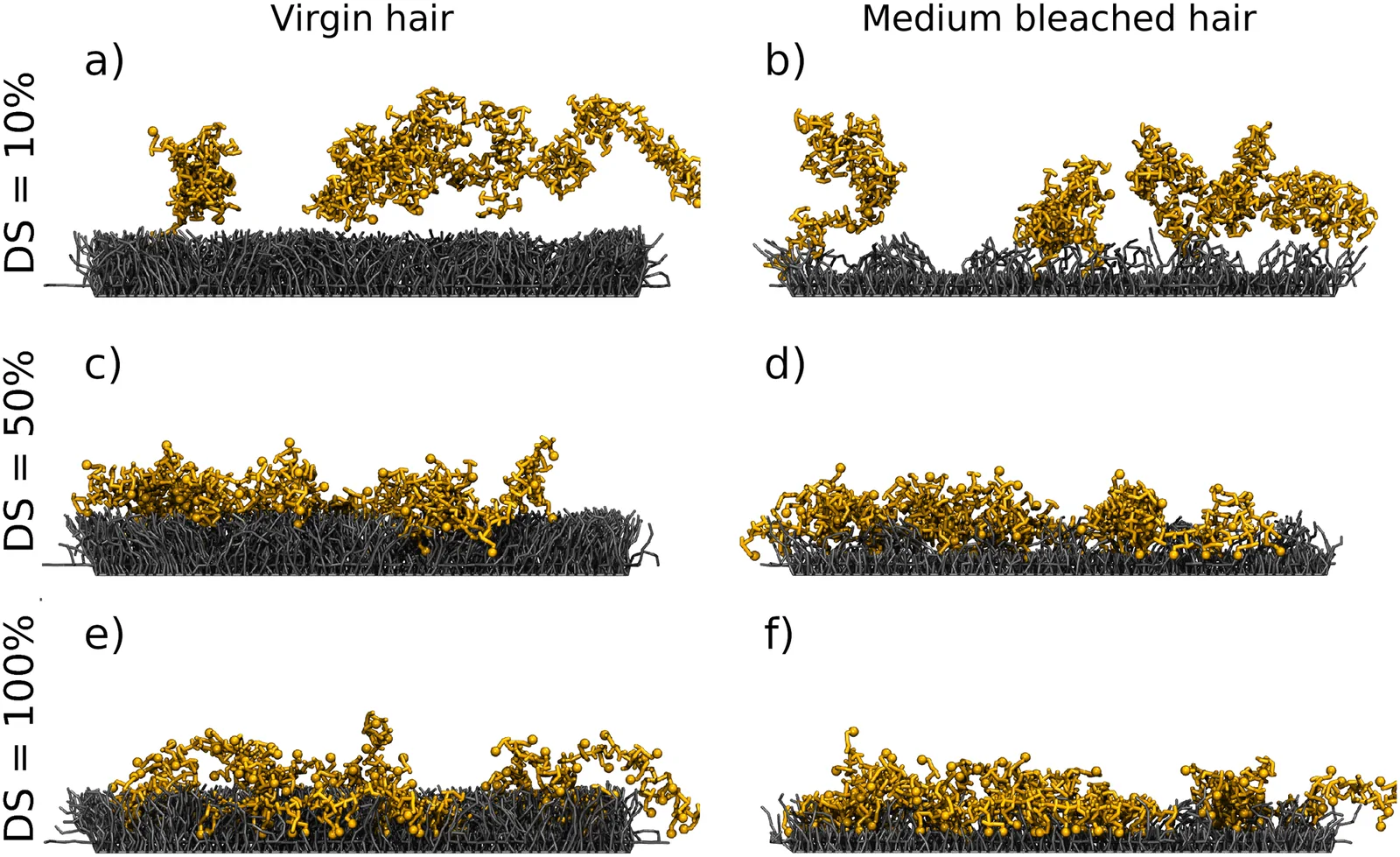
Snapshots of cationic guar gum, CGG, (orange) at various charge densities, DS = 10–100%, at the end of the CGG adsorption stage on (a, c and e) virgin and (b, d and f) bleached hair. Coarse-grained hair beads are shown in grey. Water beads are not shown. Rendered using VMD.^67^

**Fig. 3:**
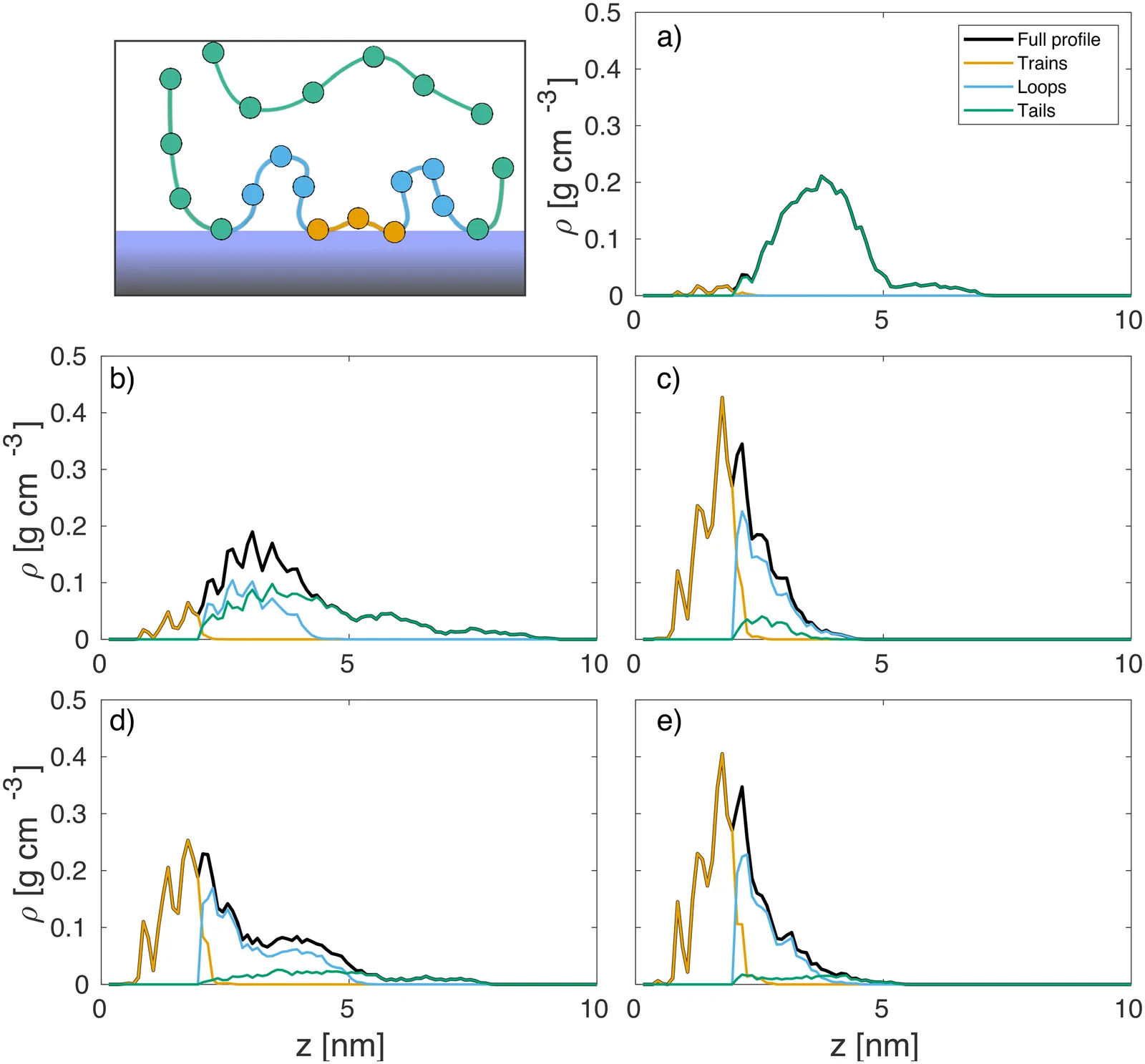
Mass density profiles for adsorbed CGG in the presence of SDS on virgin (left) and bleached hair (right) at (a) DS = 10%, (b and c) DS = 50% and (d and e) DS = 100%. The density profiles are divided into parts of CGG chains residing in trains, loops or tails.

Snapshots of CGG at different charge densities on virgin and bleached hair model surfaces at the end of the CGG adsorption stage are shown in Fig. 2. The corresponding mass density profiles are shown in Fig. 3. The overall mass density profiles were subdivided into trains lying flat on the surface, loops and tails.^12,50^ Tails are either polymers beads only bound by one adsorbed site (literal tails), or, by definition, entire polymers residing on top of other polymers (multilayers). This decomposition is based on the definitions of the adsorbed polymer structures proposed by Scheutjens and Fleer.^68^ This method has proven to be successful in other recent CG-MD studies of polymer adsorption to biomimetic hair surfaces.^42,43^ The automated classification was performed by a recursive walk across polymers, where loops are classified as parts of the polymers between two adsorbed backbone beads.

For DS = 10%, CGG only weakly adsorbs on bleached hair surfaces, primarily as tails. The DS = 10% CGG does not adsorb onto virgin hair surfaces and all the CGG chains are removed from the virgin hair contact during the later squeeze-out simulations. For bleached hair contact, a partial squeeze-out of DS = 10% CGG is observed. The CGG depletion on virgin hair indicates that polyelectrolytes with DS = 10% are not effective in providing a conditioning effect on virgin hair when deposited by itself, or sequentially with SDS.

Conversely, CGG strongly adsorbs for DS = 50% (case II) and 100% (case III). Fig. 3 shows that the CGG film thickness decreases somewhat with increasing DS, which is consistent with previous experimental studies.^47^ At DS = 100%, a higher fraction of the polymers (40%) reside in flat train conformations in the case of virgin hair. For bleached model surfaces, the fraction of train conformations is similar (57%) for DS = 50% and 100%. This observation is also in good agreement with previous QCM-D and XPS experiments of cationic polyelectrolytes with different charge densities absorbing onto hydrophobic gold surfaces.^69^ Highly charged polymers were observed to deposit in a flat conformation, while more weakly charged functionalizations adsorbed in a more extended structure.^69^

**Fig. 4:**
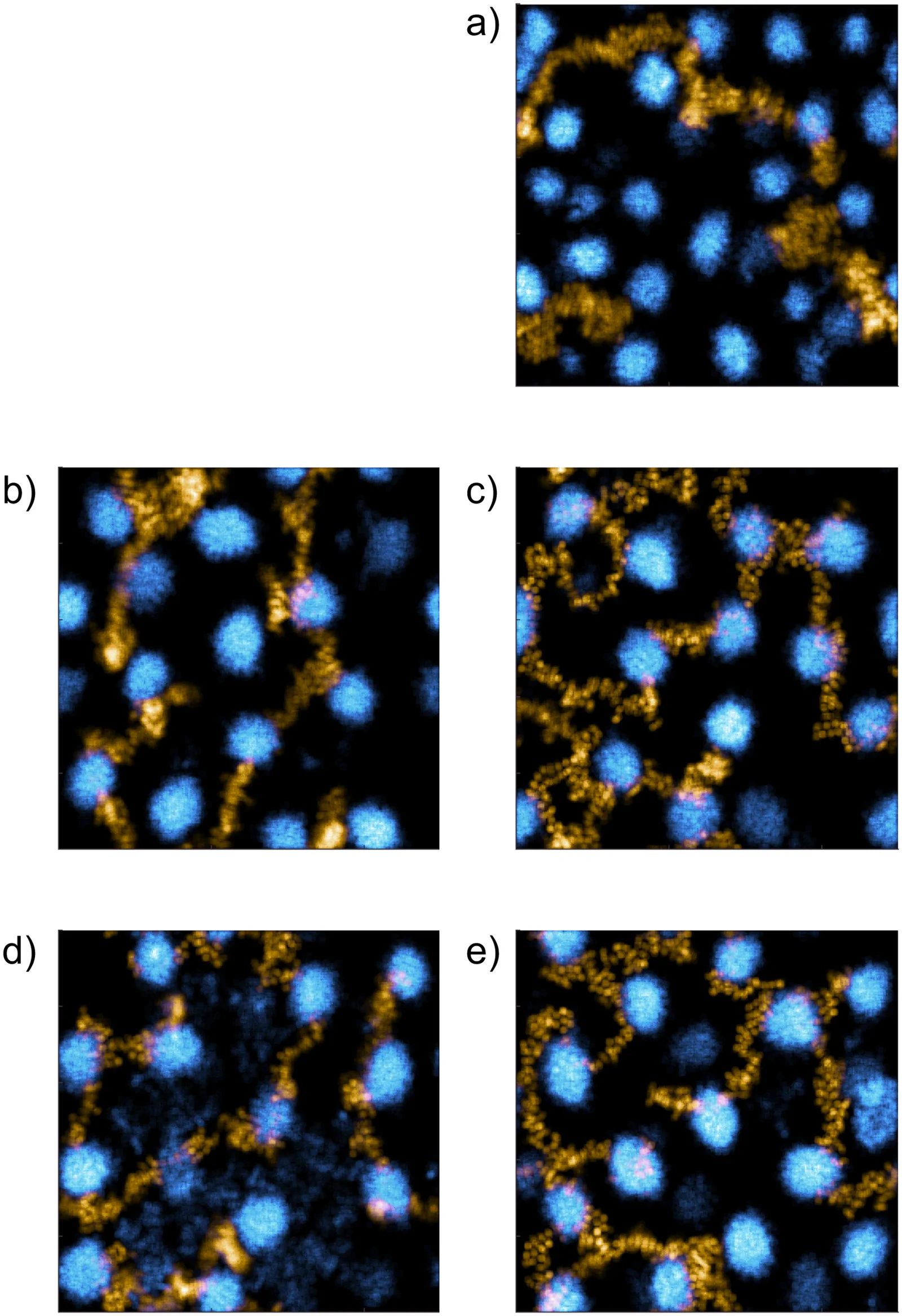
Two-dimensional coverage maps (top view) for adsorbed CGG (orange) in the presence of SDS (cyan) on virgin (left) and bleached hair (right) at (a) DS = 10%, (b and c) DS = 50% and (d and e) DS = 100%. The color intensity corresponds to a relative magnitude in local adsorption density.

The two-dimensional (*x*–*y*) distribution of CGG chains and SDS micelles on single hair surfaces is shown in Fig. 4 at different DS. For SDS, we only show molecules that are located in the vicinity of the hair surface, *z* ≤ 8 nm away from the base of the hair surface. For the more strongly charged CGG chains (DS ≥ 50%), the polyelectrolyte chains adsorb in a flatter conformation that resembles a self-avoiding random walk. The adsorbed conformation is consistent with experiments of polyelectrolyte adsorption on oppositely-charged mica surfaces.^70^ From the top view, SDS micelles are relatively evenly distributed over the plane of the surface. The SDS micelles remain mostly intact upon adsorption to the CGG and result in pearl-necklace structures.^23^ The formation of these structures is due to the hydrophobic cohesive forces within the micelles, which dominate over the attractive electrostatic forces between single cationic sites of CGG and the SDS micelles. This patchy globular structure resembles that observed experimentally for cationic polysaccharide–SDS complexes.^71^

### Shear simulations

**Fig. 5:**
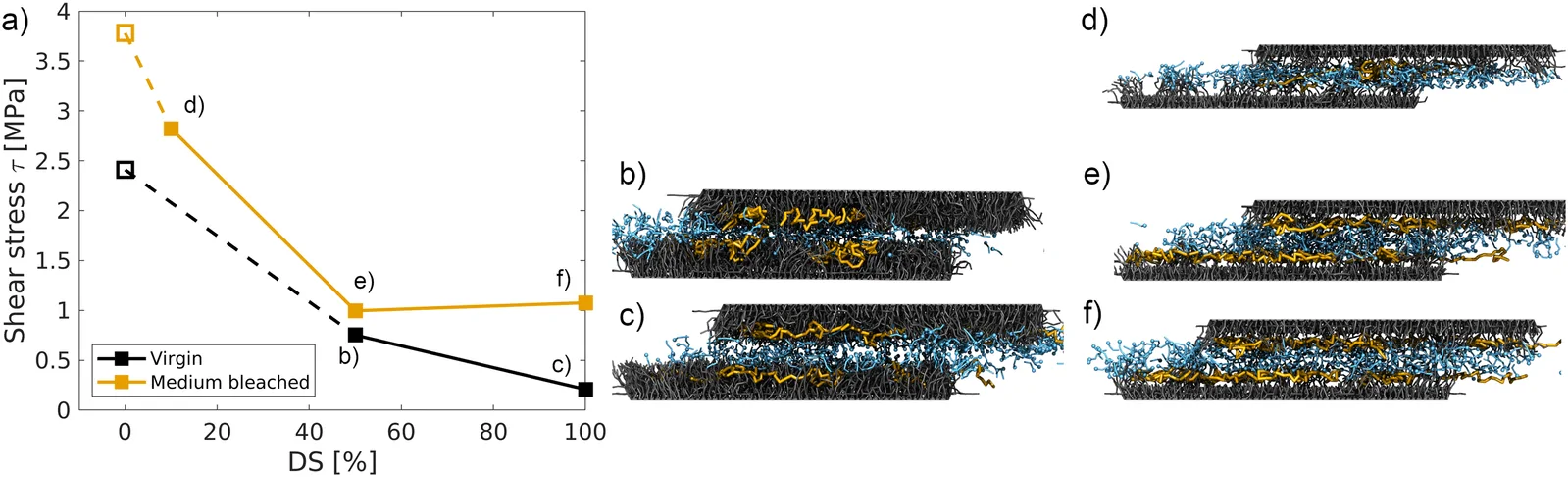
(a) Shear stress from NEMD of different cationic guar gum, CGG, charge densities (DS) with anionic surfactants, SDS, on (b and c) virgin and (d–f) bleached hair at *σ* = 10 MPa and *v*_s_ = 0.1 ms^−1^. No shear stress data is available for virgin hair at DS = 10%. The shear stress at DS = 0 represents bare hair surfaces without any CGG or SDS. The dotted lines therefore only serve as guide to the eye. Water beads are not shown. Rendered using VMD.^67^

NEMD simulations were run at a baseline pressure of *σ* = 10 MPa and a sliding velocity of *v*_s_ = 0.1 ms^−1^.^41^Fig. 5 shows the shear stress for the three levels of CGG charge density on virgin and bleached hair surfaces. No results are presented for the low-charge density CGG on virgin hair because all of the chains were removed from the contact during squeeze-out. On virgin hair, friction is reduced by moving from DS = 50% to DS = 100%. The corresponding mass density profiles for virgin and bleached hair at different DS are shown in Fig. S1 the SI. The contact composition and distribution of CGG loops, trains and tails is summarized in Table 2. The CGG mass densities divided into loops, trains and tails for different DS are provided in Fig. S2 in the SI. On virgin hair, the lower CGG charge density leads to moderate reductions in SDS (*ρ*_SDS_ = 0.97 nm^−2^) and water content (*ρ*_w_ = 13.5 nm^−2^) compared to the baseline case at DS = 100% (*ρ*_SDS_ = 1.11 nm^−2^, *ρ*_w_ = 16.9 nm^−2^). According to previous studies, CGG–SDS complexes have 2–4 surfactant molecules per cationic site and around 95% bound water.^47,72^ In our simulations, the ratio of SDS molecules to cationic headgroups on CGG is comparable to this range, suggesting that excess unbound SDS is depleted during squeeze-out.

Contact compositions and CGG properties used in the NEMD simulations (after squeeze-out) of the variable charge density systems on virgin and bleached hair surfaces

**Table:** 

Virgin hair	DS =	10%	50%	100%
Coarse-grained water	*ρ*_w_	n/a	13.5 nm^−2^	16.9 nm^−2^
SDS	*ρ*_SDS_	n/a	0.97 nm^−2^	1.11 nm^−2^
SDS molecules per cat. site	*n* ^−^/*n*^+^	n/a	3.21	1.82
CGG loops : trains : tails	l : tr : tl	n/a	0.34 : 0.60 : 0.06	0.26 : 0.73 : 0.01

**Table:** 

Med. bleached hair	DS =	10%	50%	100%
Coarse-grained water	*ρ*_w_	19.0 nm^−2^	21.5 nm^−2^	20.9 nm^−2^
SDS	*ρ*_SDS_	0.74 nm^−2^	0.86 nm^−2^	0.87 nm^−2^
SDS molecules per cat. site	*n* ^−^/*n*^+^	12.12	2.82	1.43
CGG loops : trains : tails	l : tr : tl	0.44 : 0.55 : 0.01	0.09 : 0.91 : 0.00	0.11 : 0.89 : 0.00

As a result of the lower charge density, the separation between the opposing 18-MEA monolayers is considerably reduced. A single peak in the SDS distribution between the surface (see Fig. S1 in the SI) suggests that the SDS molecules are more disordered than at higher DS. Negligible interdigitation is observed between the CGG polyelectrolytes adsorbed onto the opposing surfaces, which is in agreement with the high-charge density results.^41^

On bleached hair, the low-charge density CGG also adsorbs *via* only a few charged sites on the surfaces, as evident from Fig. 2. This leads to a partial squeeze-out and a CGG adsorption density of *Γ* ≈ 0.02 μg cm^−2^, which corresponds to two polysaccharides per surface. Surface area densities of *ρ*_SDS_ = 0.74 nm^−2^ and *ρ*_w_ = 19.0 nm^−2^ are found for SDS and water at DS = 10%. The number of SDS molecules in relation to CGG cationic sites is much higher than for virgin hair or higher DS. This suggests that either physical adsorption to the few remaining 18-MEA patches or physical adsorption/trapping by the CGG molecules takes place.

At a DS of 50%, all the CGG molecules remained inside the contact during squeeze-out. The corresponding SDS and water area densities within the contact are *ρ*_SDS_ = 0.86 nm^−2^ and *ρ*_w_ = 21.5 nm^−2^ respectively. This is nearly identical to the values for the fully substituted CGG polymers reported in our previous study.^41^ Large friction reductions on bleached hair are observed between DS = 10–50%, while friction is nearly constant in the range of DS = 50–100%. The initial friction reductions can be explained by an increase of CGG and SDS inside the contact and by the asymmetric slip of the remaining CGG polyelectrolytes in the low-charge density contact, as evident from the snapshot in Fig. 5(d). Due to the low surface separation, CGG can temporarily adsorb on both surfaces simultaneously. During sliding, the molecule is then stretched in the direction of sliding. It eventually desorbs on one side of the contact, which is expected to create strong dissipative contributions as a secondary slip plane is introduced. Nonetheless, the shear stress at low CGG charge density is somewhat decreased compared to the untreated model hair surface (−24%). This shows that low CGG adsorption densities (DS = 10%) coupled with weak binding of SDS is still moderately effective at reducing hair friction, although this cannot fully recover the lubrication performance of virgin hair for bleached hair contacts.

At CGG degrees of substitution between 50% and 100%, the structure of the complex in bleached hair contacts does not change considerably, with similar degrees of water and SDS content confined within the contact during sliding. Compared to virgin hair, more cationic CGG sites adsorb on the surface, which reduces the available cationic sites for SDS micelle adsorption. This could explain why the shear stress decreases for virgin hair but does not change significantly for bleached hair at high DS. Compared to the untreated bleached hair contacts, the shear stress is reduced by 73% at DS = 50%. These results indicate that a DS of 50% is indeed sufficient to obtain considerable improvements in the lubrication performance on bleached hair at the given adsorption densities.

### Effect of the polymer adsorption density

Next, we investigated the effect of the CGG adsorption density on SDS deposition, squeeze-out and hair friction. In the baseline simulations presented in our previous study,^41^ the majority of negative surface charges was balanced by adsorbed counterions rather than cationic polysaccharides. This is a result of complete adsorption of the few polymers available in the finite-size simulation bulk. Experimental QCM-D adsorption studies by Guzmán *et al.*^44^ of similar cationic polysaccharides (JR400) suggest a threefold increase in adsorption densities on biomimetic charged layers of a sulfonic acid salt compared to uncharged thiols. A strong increase in adsorption density of cationic surfactants on bleached hair compared to virgin hair was also observed in earlier CG-MD simulations.^45^ It is therefore reasonable to assume that bleaching will increase the affinity and equilibrium adsorption density of larger cationic polysaccharides such as CGG.

We therefore conducted simulations with increased guar adsorption densities of *Γ* = 0.12 μg cm^−2^ on bleached hair surfaces. This is similar to the adsorption densities reported for JR400 on functionalized, negatively-charged surfaces due to Guzmán *et al.*^44^ Virgin hair surfaces are also considered at this adsorption density for completeness but may be of less relevance, since the adsorption is expected to be closer to the original value used in our previous study (*Γ* = 0.04 μg cm^−2^).^41^

### Adsorption on single surfaces

**Fig. 6:**
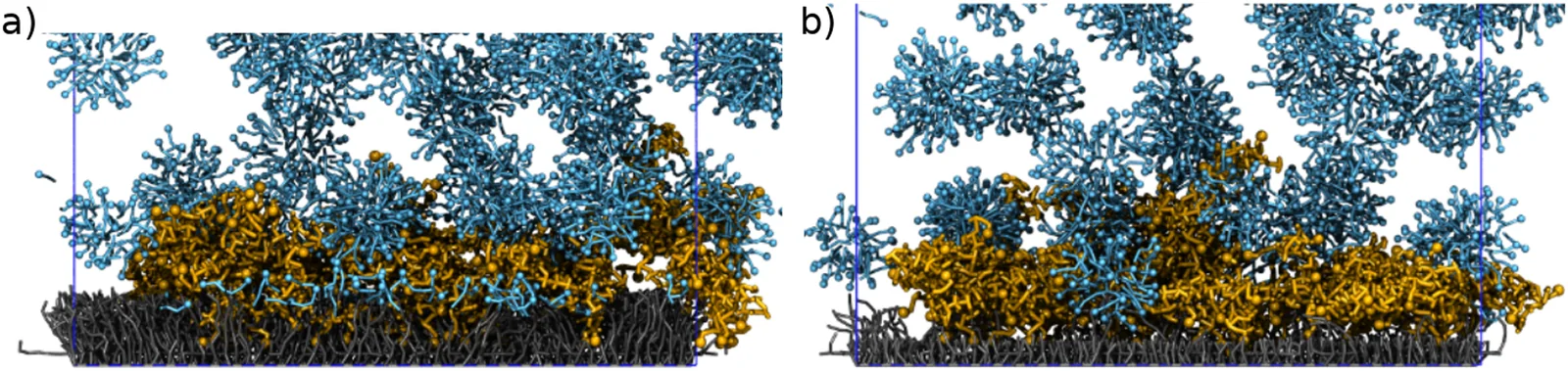
Snapshots of sequentially adsorbed cationic guar gum, CGG, at *Γ* = 0.12 μg cm^−2^ (orange) and SDS (cyan) at the hair interface (grey) for (a) virgin and (b) bleached hair surfaces. Water beads are not shown. Rendered using VMD.^67^

Snapshots of the adsorbed structures on a single model hair surface are shown in Fig. 6. The corresponding snapshots of adsorbed CGG before introducing SDS are shown in Fig. S3 in the SI. Upon addition of an SDS bulk, SDS micelles migrate to the surface and electrostatically adsorb to the cationic sites on the polysaccharide layer. In the presence of SDS, the CGG layer thickness on virgin and bleached hair surfaces is 6.1 nm and 6.0 nm respectively. Previous experiments of oppositely charged polymer–surfactant structures on hydrophilic surfaces^73^ also reported swelling of the adsorbed networks, which could provide brush-like conformations with favorable lubrication properties.^27,28^ The degree of swelling of CGG when exposed to the SDS bulk is shown in Fig. S4 in the SI. Negligible changes in the surface-normal mass density profiles are observed even at relatively long times (*t* > 70 ns) in the present configurations. This might be due to the relatively high charge density of the polysaccharides (DS = 50%) resulting in a high number of electrostatically adsorbed quaternary ammonium groups and strong attractive forces to the substrate. Longer polymers, increased adsorption densities, or reduced CGG charge densities might be required to observe this effect. Longer polymers have indeed been found to show a weaker friction force coupling with sliding velocity in recent SFA experiments on hair mimic surfaces with chitosan polymers, even in the absence of surfactants.^28^ On the other hand, Qian *et al.*^74^ previously observed that the addition of anionic surfactants led to a more condensed layer of adsorbed cationic polyelectrolytes on mica. According to Fig. S4 in the SI, we find that the layer thickness of the polyelectrolyte does not decrease either due to the addition of SDS. The CGG chains studied in this work show high stability against swelling or condensation within the observable time scales.

It is also worth noting that no direct entropy-driven counterion release was observed here from either CGG or SDS micelles. This mechanism has been observed during coacervation in both experiments^12^ and coarse-grained bulk simulations.^34^ A systematic variation of the background ionic strength could be appropriate to probe for this mechanism in future simulation studies. One reason for why this mechanism is not observed could be that counterion release might require much longer simulation times than the ones accessed in the current simulations.^75^ It was also shown that the MARTINI 3 force field could capture counterion release for an oppositely charged polyelectrolyte–polyelectrolyte complex over microsecond timescales.^34^ We therefore cannot rule out any intrinsic underestimation of the phenomenon with the MARTINI 2 parameters used here.

**Fig. 7:**
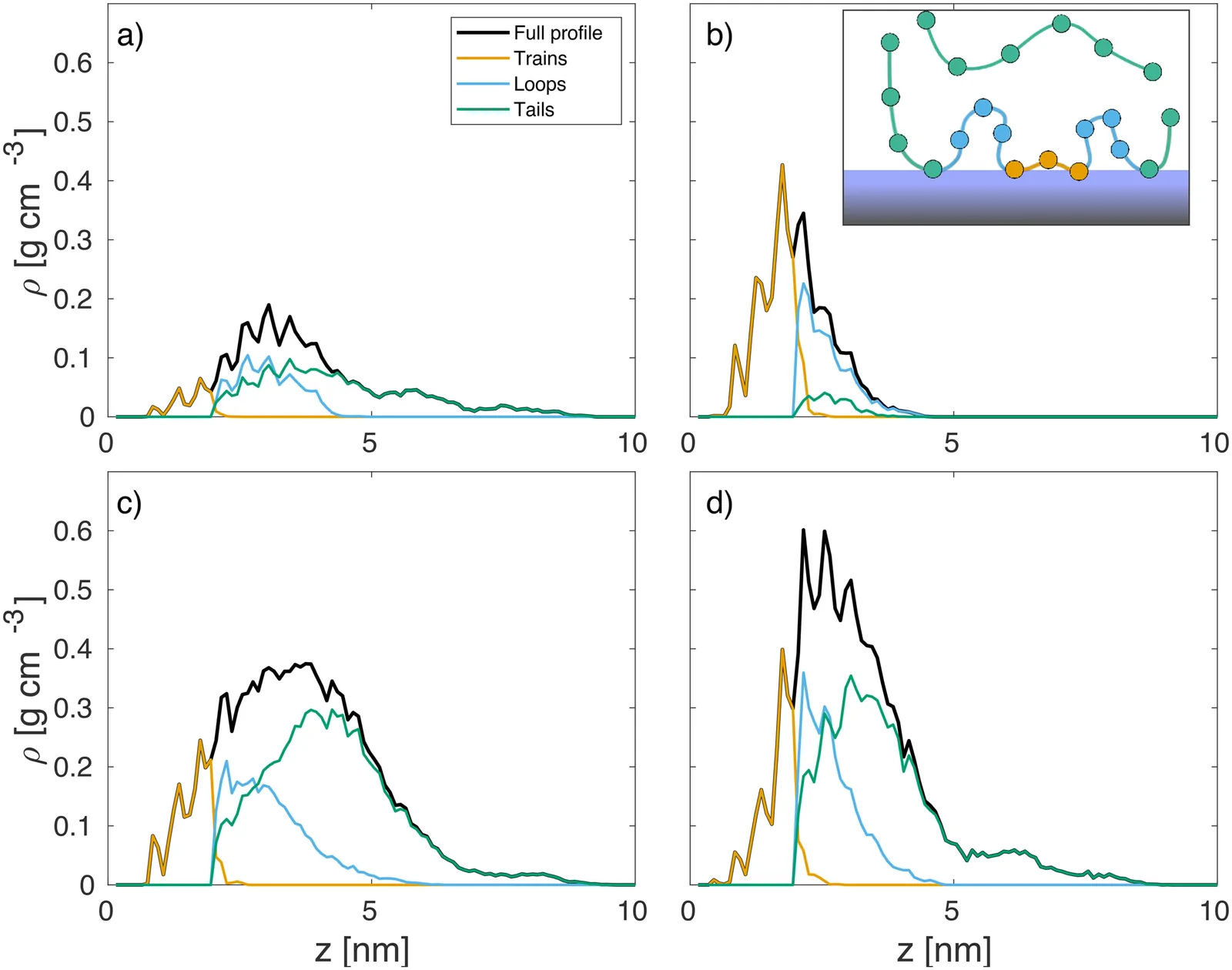
Mass density profiles for adsorbed CGG in the presence of SDS on virgin (left) and bleached hair (right) at CGG adsorption densities of (a and b) *Γ* = 0.04 μg cm^−2^ and (c and d) *Γ* = 0.12 μg cm^−2^. The CGG density profiles are decomposed into trains, loops or tails.

Time-averaged mass density profiles of CGG in the presence of SDS at the end of the adsorption stage are shown in Fig. 7 for low (case II) and high (case IV) CGG adsorption densities. The mass density profiles were again divided into trains, loops and tails.^68^ The observed profiles are similar to previous CG-MD simulations of hydrophilic flexible linear homopolymers adsorbing onto heterogeneous biomimetic hair surfaces.^42^ Here, the highest contribution to flat trains on the surface is observed on bleached hair and high CGG adsorption densities. This is a result of the high number of anionic sites and increased number of cationic charged on the polyelectrolytes. Tails account for the majority of adsorbed CGG in all cases except for low CGG adsorption densities on bleached hair, where loops become the dominant contribution. In that case, CGG forms a thin layer with few tails extending into the liquid because of the strong electrostatic adsorption onto the surface. For virgin hair at low CGG adsorption densities, the comparably weaker adsorption leads to single CGG chains protruding into the liquid consistent with the snapshot shown in Fig. 2c. For high CGG adsorption densities, a large fraction of polyelectrolyte material is adsorbed on top of other CGG chains, forming multilayers. These multilayers can form at sufficiently high (local) ionic strength and are driven by hydrophobic interactions between the uncharged fragments of the polymer chains.^12^ The upper layers in these aggregates are bound by relatively weak hydrophobic interactions with other polymers, which is enhanced by electrostatic interactions with intercalating SDS micelles. During the squeeze-out stage, these multilayers resisted lateral depletion, which suggests that the polymer–polymer interactions are sufficiently strong to remain stable at these pressures. Further investigations might be necessary to investigate whether this holds also true for thicker polymer layers and higher pressures.

**Fig. 8:**
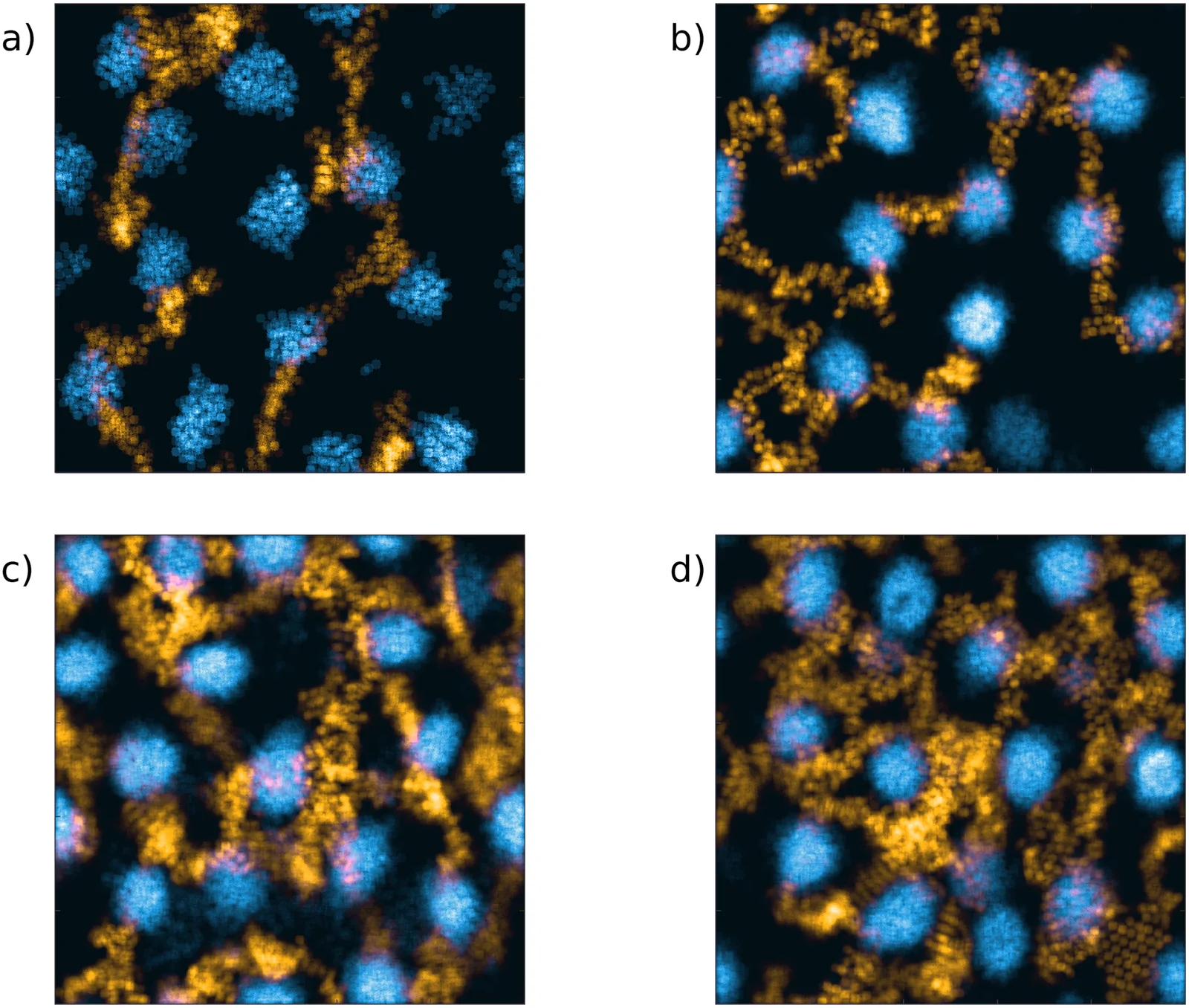
Two-dimensional coverage maps (top view) for adsorbed CGG (orange) in the presence of SDS (cyan) on virgin (left) and bleached hair (right) at (a and b) *Γ* = 0.04 μg cm^−2^ and (c and d) *Γ* = 0.12 μg cm^−2^. The color intensity corresponds to a relative magnitude in local adsorption density.

We show the local distribution of CGG chains and SDS micelles on single hair surfaces in Fig. 8 at the two respective CGG adsorption densities. The same *z*-distance cutoff of 8 nm was used for the shown SDS molecules. SDS is organized in micelles that remain evenly distributed for different CGG adsorption densities. There is no clear correlation between the organization of micelles and CGG deposition from the top view. However, as evident in Fig. 6, SDS micelles near the hair surface indeed adsorb onto the cationic CGG sites, thus forming pearl-necklace structures.^23^ The repulsive interactions between negatively-charged SDS micelles could explain the more even distribution as opposed to the more localized CGG chains. This might in turn facilitate a more even distribution of anionic surfactants under load and shear, which will be studied next.

### Shear simulations

**Fig. 9:**
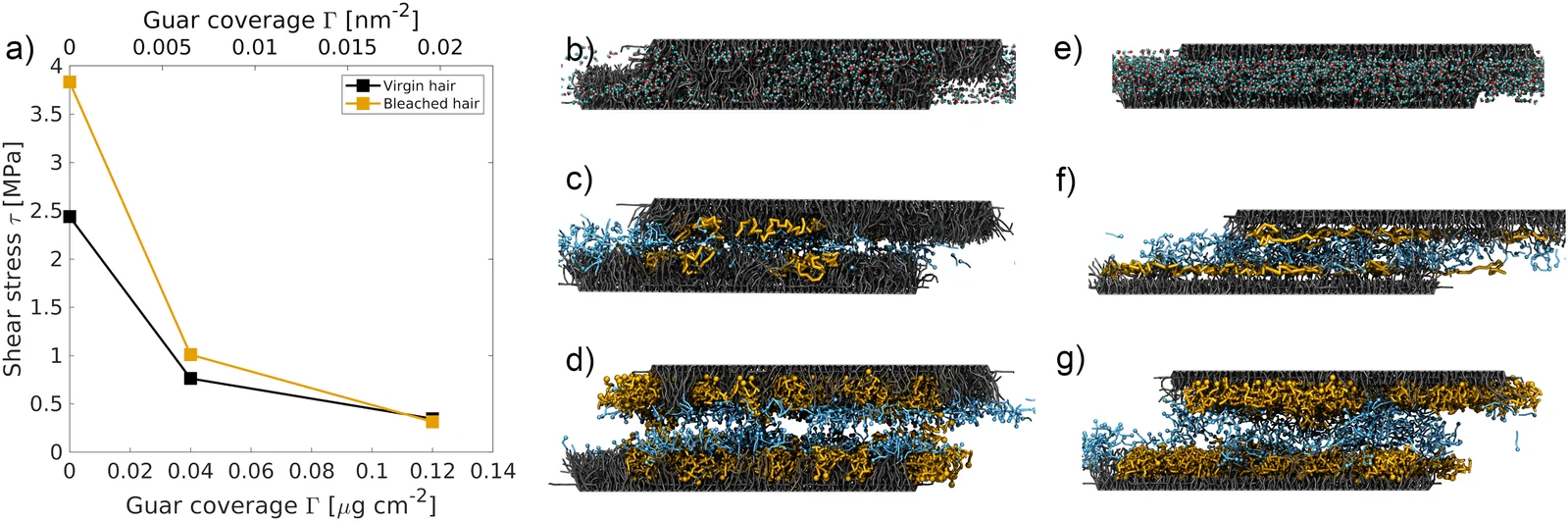
(a) shear stress as a function of the CGG mass-based (bottom axis) and area-based (top axis) adsorption density with anionic surfactants, SDS, for (b–d) virgin and (e–g) bleached hair at *v*_s_ = 0.1 ms^−1^ and *σ* = 10 MPa. Water beads are not shown. Rendered using VMD.^67^

The shear stress for virgin and bleached hair as a function of the CGG adsorption density (*Γ* = 0–0.12 μg cm^−2^) with SDS is shown in Fig. 9. The shear stress decreases non-monotonically with increased CGG coverage on both virgin and bleached hair. While the shear stress is generally lower on virgin hair than bleached, friction becomes less sensitive to the degree of hair damage at high CGG coverage. The simulation snapshots show that the separation between the two hair surfaces increases for the systems with higher CGG coverages.

**Fig. 10:**
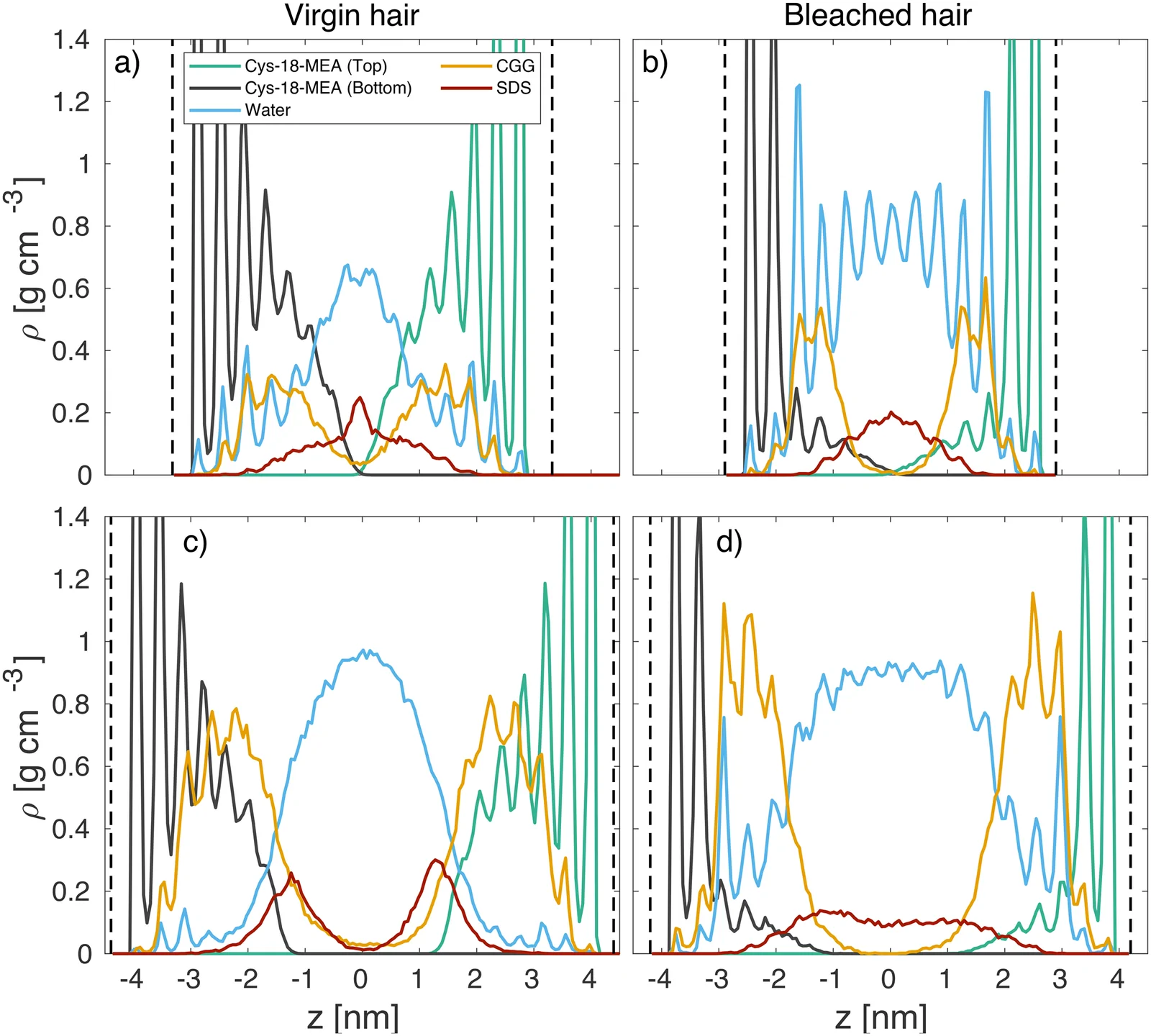
Through-film mass density, *ρ* for virgin (a and c) and bleached hair (b and d) at guar adsorption densities of (a and b) *Γ* = 0.04 μg cm^−2^ and (c and d) *Γ* = 0.12 μg cm^−2^.

Fig. 10 shows the structure of the contacts at the two considered CGG adsorption densities. The mass density profiles suggest full surface separation for both virgin and bleached hair by means of a thick water layer. As with our previous simulations,^41^ SDS is hydrophobically adsorbed on top of the CGG polymers and 18-MEA on virgin hair, allowing for higher water uptake during squeeze-out. A summary of the area densities of water and SDS for the two considered levels of CGG adsorption is given in Table 3. The area density of SDS (*ρ*_SDS_ = 1.45 nm^−2^) in the virgin hair contact is increased compared to the baseline case with a lower CGG coverage. At the same charge density, larger polymers offer more cationic sites to bind SDS molecules and retain them inside the contact even under compressive loads. An increase in water area density (*ρ*_w_ = 22.9 nm^−2^) is also observed compared to the baseline case.

Contact compositions and CGG properties used in the NEMD simulations (after squeeze-out) of the variable coverage systems on virgin and bleached hair surfaces

**Table:** 

Virgin hair	*Γ*_CGG_ =	0.04 μg cm^−2^	0.12 μg cm^−2^
Coarse-grained water	*ρ*_w_	13.5 nm^−2^	22.9 nm^−2^
SDS	*ρ*_SDS_	0.97 nm^−2^	1.45 nm^−2^
SDS molecules per cat. site	*n* ^−^/*n*^+^	3.21	1.19
CGG loops : trains : tails	l : tr : tl	0.34 : 0.60 : 0.06	0.41 : 0.44 : 0.15

**Table:** 

Med. bleached hair	*Γ*_CGG_ =	0.04 μg cm^−2^	0.12 μg cm^−2^
Coarse-grained water	*ρ*_w_	21.5 nm^−2^	33.4 nm^−2^
SDS	*ρ*_SDS_	0.86 nm^−2^	1.23 nm^−2^
SDS molecules per cat. site	*n* ^−^/*n*^+^	2.82	1.05
CGG loops : trains : tails	l : tr : tl	0.09 : 0.91 : 0.00	0.32 : 0.65 : 0.03

Two primary peaks in the SDS mass density profiles suggest that the surfactants remain adsorbed on CGG under shear in the virgin hair contact. Micelle break-up is observed with SDS maintaining a reverse-bilayer structure.^76^ The majority of CGG polymers are confined between the covalently bound 18-MEA layer on virgin hair under compression. The water content in this region is considerably reduced compared to the contact center, which is probably a result of stronger hydrophobic forces due to compression. It is possible that compression-induced dehydration of the CGG layers contributes to a thicker water layer at the contact centre. The addition of SDS did not affect the CGG layer thickness notably. To quantify whether squeeze-out or shear is responsible for this conformational change compared to the single surfaces, we compared the mass density profiles at three points along the simulation process: (1) before squeeze-out, (2) before the beginning of the NEMD and (3) at dynamic equilibrium in the NEMD simulations. The corresponding mass density profiles on virgin and bleached hair are shown in Fig. S5 and S6 in the SI. The mass densities suggest that the conformational change occurs during compression of the two surfaces in the squeeze-out step.

For bleached hair, SDS is relatively evenly distributed in the contact center and does not display any surface-normal ordering. The area density of SDS (*ρ*_SDS_ = 1.23 nm^−2^) and water (*ρ*_w_ = 33.4 nm^−2^) are increased compared to the baseline case at lower CGG adsorption density. The shear stress is comparable to that of virgin hair at the same CGG adsorption density. Therefore, this structural change between the different surfaces by itself cannot fully explain the observed friction reductions across the different degrees of damage. Furthermore, the water depletion from the CGG-dense region in the bleached hair contact is less pronounced than for virgin hair. This is because of the compression-induced intercalation of CGG with the dense 18-MEA monolayer on virgin hair, which is mostly removed on bleached hair. This also means that the central water film is probably not promoted due to compression-induced dehydration of the CGG layer, as hypothesized earlier.

**Fig. 11:**
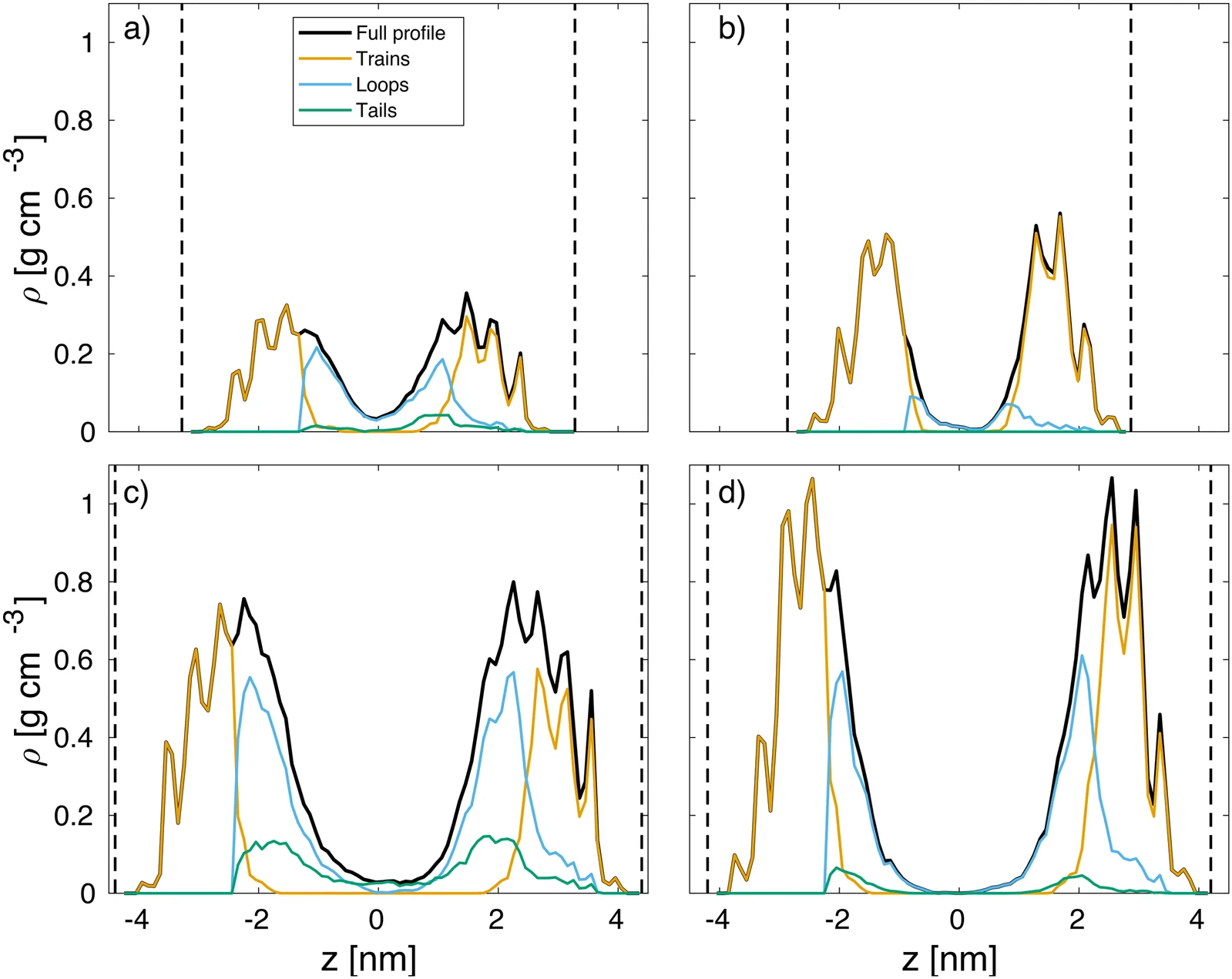
Time-averaged mass density profiles of CGG decomposed into trains, loops and tails during NEMD sliding simulations. Profiles are shown for virgin (left) and bleached hair (right) at (a and b) *Γ* = 0.04 μg cm^−2^ and (c and d) *Γ* = 0.12 μg cm^−2^.

One mechanism that could explain the lower friction at higher CGG adsorption densities is connected to the higher contributions of loops to the mass density profiles of CGG on the surfaces, as shown in Fig. 7. This mechanism has also been highlighted in a previous study involving hair combing experiments using polyelectrolyte–surfactant containing conditioners.^25^ To quantify this, we further plotted the distribution of tails, loops and trains to the mass density profiles of CGG inside the sliding contact during the NEMD simulations, as shown in Fig. 11. We indeed observe a higher proportion of loops at higher CGG adsorption density. However, even at low CGG coverage, there is only a small amount of interdigitation between the CGG films adsorbed on the opposing surfaces, as shown by the near-zero density in the centre of the contact. Since the CGG films remain well-separated, we do not expect their conformation to directly affect the lubrication between the two surfaces. This polymer-brush effect^27,29^ may become more significant at the higher pressures studied in our previous work,^41^ where the CGG layers adsorbed to opposing sliding surfaces are more likely to come into direct contact. It should also be noted that our simulations use smooth substrates, whereas real hairs possess microscale roughness features, particularly at the cuticle edges, which significantly affect friction^77^ and formulation deposition (*e.g.*, meniscus effect at the cuticle edge).^78^ Thus, the polymer-brush effect might be more relevant to reduce friction in a roughness-induced boundary lubrication regime and eliminate stick–slip, as observed for other formulations.^79^ Explicit modelling of micron-sized roughness features is not feasible with the simulation techniques used in this work and would require the use of multiscale techniques.^80^ However, the present methods are deemed reliable to predict qualitative changes in the average friction of the exposed epicuticle surface, which accounts for the majority of the surface area in human hair.^80^

As in our previous simulations,^41^ we observe synergistic lubrication performance between SDS and CGG, with friction of the adsorbed complex being much lower than for either individual component.^81^ This observation is consistent with previous AFM colloidal probe experiments, which showed that the presence of SDS was required to obtain notable friction reductions when present on mica surfaces with adsorbed cationic polyelectrolytes.^82^ Similarly, synergistic drag reductions have also been observed in shear MD simulations of guar–surfactant complexes at the liquid–vapor interface.^83^ The addition of SDS to CGG was also found to further reduce hair friction in experiments.^26^ When used without CGG, the anionic SDS micelles are easily squeezed out of the contact due to electrostatic repulsion with the hair surfaces, while without SDS, CGG chains interdigitate with those adsorbed to the opposing surface, which leads to high friction.^41^ When used together, CGG strongly adsorb on the anionic hair surfaces and traps SDS molecules inside the contact.^41^ The trapped SDS molecules provide a direct lubrication effect and increase the amount of water trapped within the contact, which also reduces friction.^41^ Our current simulations predict that SDS adsorption increases before squeeze-out (+28% for virgin, +37% for bleached) with higher CGG adsorption densities, due to the higher number of available cationic sites on the polymer. On average, more than one surfactant adsorbs per cationic site on CGG, which is due to micellar adsorption. The SDS micelles collapse during the squeeze-out simulations and reorient to form a reverse-bilayer structure.^76^ This structure traps water molecules at the sliding interface, reduces interdigitation between the CGG chains on the opposing surfaces, and lowers friction. The increased amount of SDS and water trapped inside the contact for the higher CGG surface coverages (Table 3) and CGG charge densities (Table 2) therefore have a beneficial lubrication effect. These various contributions to the lubrication mechanism of CGG–SDS complexes with low and high DS are summarized in Fig. 13. The friction reduction provided by the CGG with higher DS can be attributed primarily to the SDS trapping mechanism and the flatter conformation of these CGG chains does not significantly affect their lubrication performance.

**Fig. 12:**
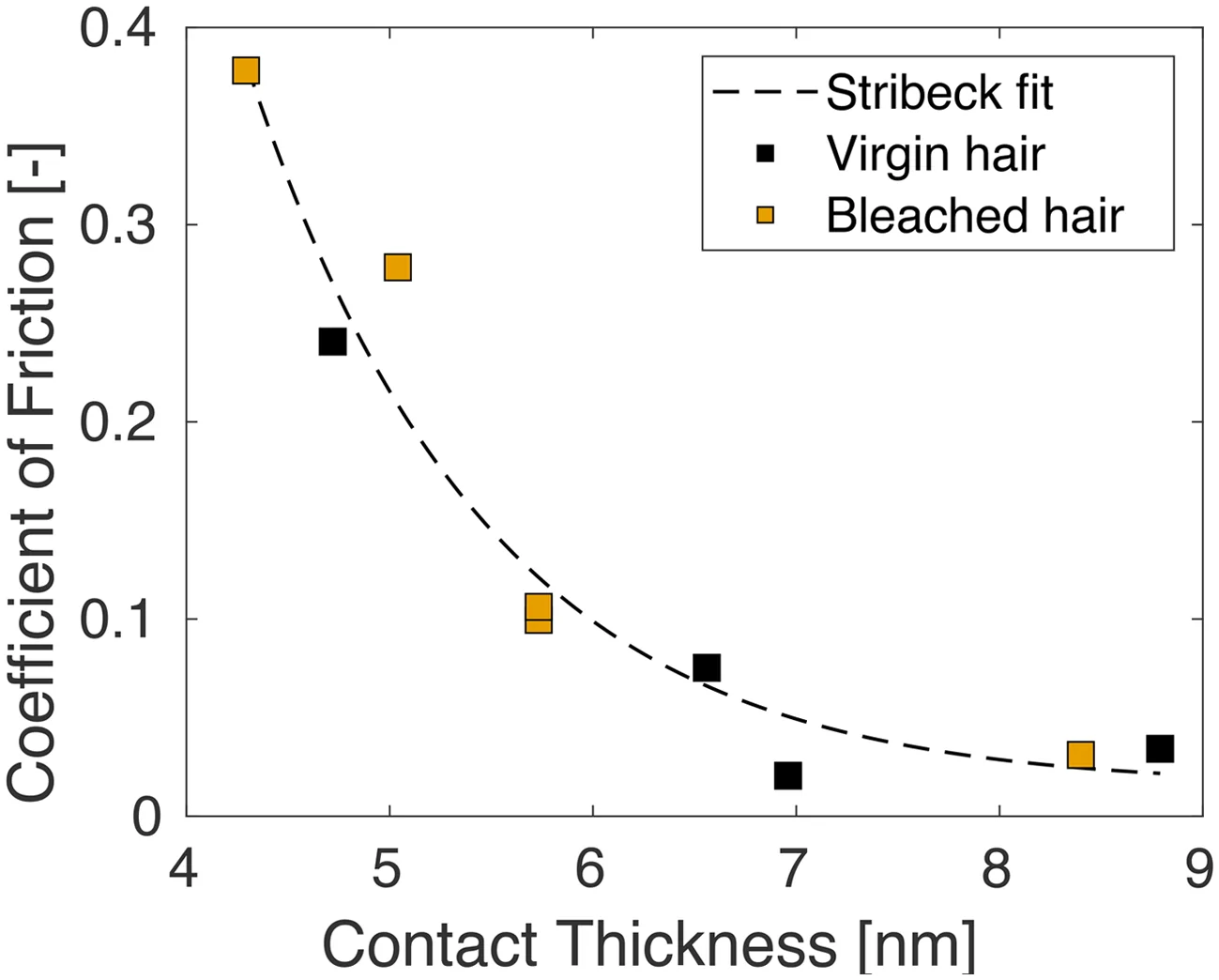
Coefficient of friction variation with overall contact thickness during sliding across different all degrees of CGG adsorption and CGG charge density (DS) considered in this work on virgin and bleached hair. A Stribeck curve exponential decay model^86^ is fitted to the data to illustrate the friction trend with contact thickness.

The strongly adsorbed polyelectrolyte–surfactant complexes push the tribological contact from the boundary lubrication regime towards the mixed and hydrodynamic regimes by locking water inside the contact. This is challenging in aqueous lubrication due to the low viscosity (and pressure–viscosity coefficient) of water compared to oils.^84^Fig. 12 shows the change in the coefficient of friction (shear stress divided by normal stress, 10 MPa) with contact thickness (measured as the separation in the *z*-direction between the anchoring graphene sheets of the two hair surfaces). It shows the expected Stribeck-curve-like behaviour,^85^ with an exponential decay in the friction coefficient with film thickness (entrainment velocity).^86^ Since the 18-MEA layers on the hair are approximately 2 nm thick,^53^ a thickness of 4 nm means that the opposing surfaces are in direct contact.^46^ The root-mean-square (RMS) roughness of our biomimetic hair surfaces is 1.77 nm for virgin and 0.94 nm for bleached,^45^ which is close to the RMS roughness of the epicuticle of real hair (1–2 nm).^87^ Therefore, a CGG–SDS film thickness of 8 nm is larger than the composite surface roughness and is therefore sufficient to move the hair–hair contact from the boundary to the mixed lubrication regime. One important distinction from conventional Stribeck curve behaviour in oil-lubricated systems is that, at physiologically relevant sliding velocities, the hydrated polyelectrolyte–surfactant films do not require hydrodynamic lift to separate the sliding surfaces. Instead, the complexes remain trapped between the surfaces due to strong electrostatic interactions with the hair surfaces. This mechanism mirrors other aqueous lubrication systems,^88^ such as surface-grafted polyelectrolyte brushes.^89^

**Fig. 13:**
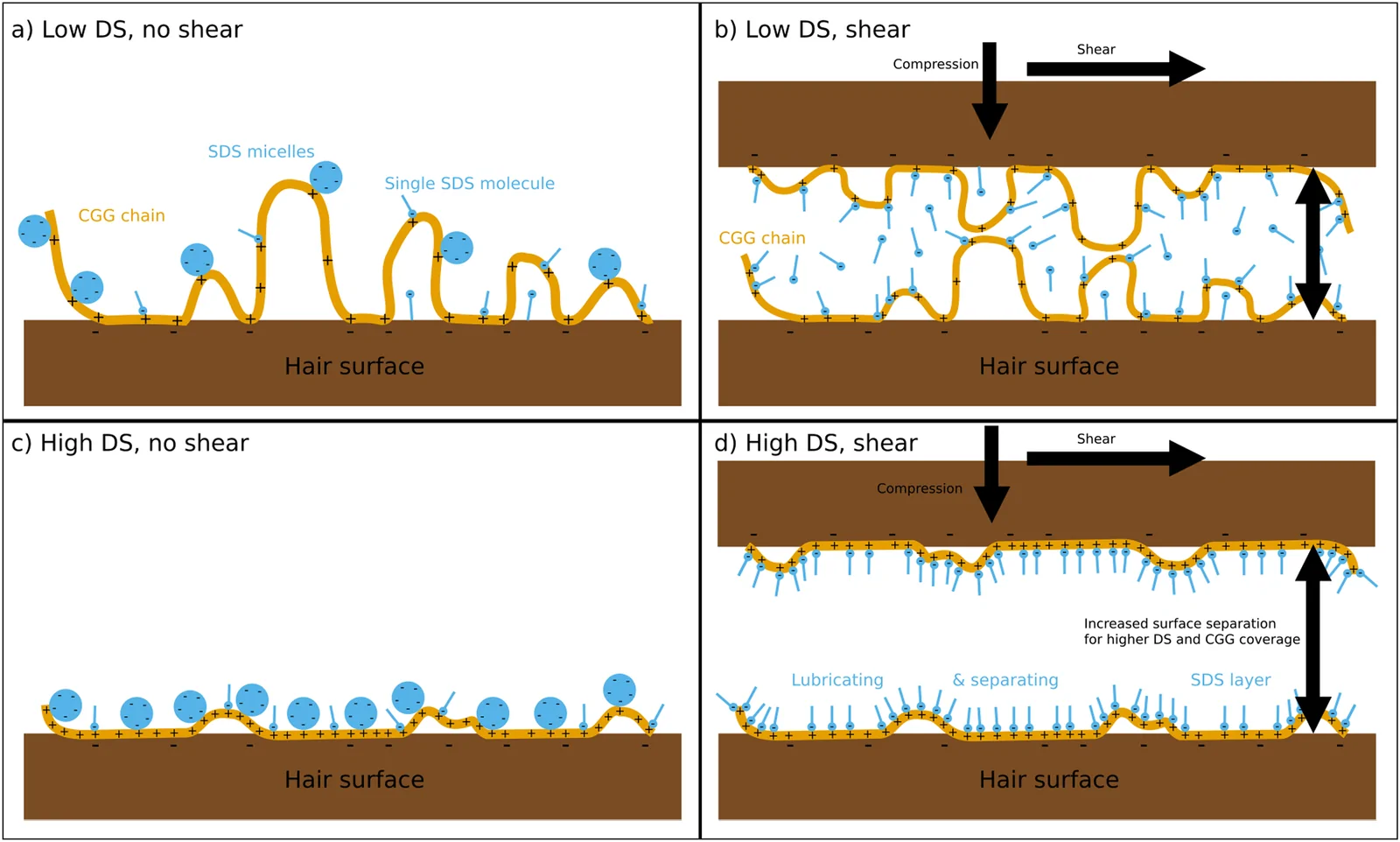
Proposed lubrication of the CGG–SDS complex at different degrees of substitution (DS). (a) Surfactant micelles and single molecules adsorb to the surface, driven by electrostatic attraction to the cationic CGG groups. (b) Compression (and shearing) breaks up the micelles and forms an SDS monolayer layer that helps to separate the CGG chains on the opposing surfaces and retains water inside the contact. (c) Increased SDS adsorption at high DS, (d) providing a lubricating SDS layer under compression and shear that prevents interdigitation between the CGG chains on the opposing surfaces. The flatter conformation of the CGG chains at higher DS (c and d) compared to lower DS (a and b) appears to have little direct effect on friction.

Table S1 in the SI provides a qualitative overview of the findings in this work with related literature experiments. To the best of our knowledge, no small-scale friction experiments of the CGG–SDS pair with hair or biomimetic surfaces exist. Studies of other polymer–surfactant pairs does not report conclusive trends for the change of film thickness with anionic surfactant addition.^74,82^ Swelling or layer collapse may be sensitive to the polymer architecture, surfactant type and other properties such as polymer charge density, molecular weight and salt concentration. Our work does not confirm either of these mechanisms with the CGG layer thickness remaining unchanged with SDS addition, as discussed earlier. Likewise, no consensus is reached regarding the capability of polymer–surfactant pairs to reduce friction. Dedinaite *et al.*^82^ and our simulations suggest friction reductions upon adding anionic surfactants to the polymer formulation. Qian *et al.*^74^ on the other hand report increased friction in SFA experiments of hydroxyethyl cellulose (HC)/SLES compared to pure HC formulations. This was attributed to a more compact conformation of the polymer due to charge neutralization by the surfactant.^74^ Conversely, a high fluidity may be required in the polymer–surfactant layer^82^ which is consistent with the observations in our simulations.

## Conclusions

In this study, we explored the effects of different levels of cationic functionalization and adsorption density of a polyeletrolyte, CGG, with respect to hair lubrication in the presence of anionic surfactants, SDS. In a first instance, lower degrees of charge density on the CGG chains compared to the fully charged baseline case were tested. During the sequential adsorption of CGG and SDS, we did not observe any notable swelling of the CGG layer upon SDS addition, contrary to previous experimental studies.^16,73^ Similarly, we did not observe any notable counterion release in our simulations. At a low degree of substitution DS = 10%, CGG chains were found to be prone to squeeze-out under physiologically relevant pressures. This is due to a reduced number of strong ionic interactions between the CGG cationic groups and anionic sites on the surface, especially for virgin hair.

Large friction reductions were observed for DS = 50% and above for both virgin and bleached hair. On the other hand, no further friction reductions were obtained from doubling the CGG charge density from DS = 50% to 100% on the bleached model hair surfaces.

The CGG adsorption density was increased to represent the amount of CGG adsorption expected on bleached hair. This resulted in further friction reductions compared to the baseline CGG case, which suggests that thicker layers of strongly charged CGG chains are effective at trapping SDS and water in a well-defined lubricating layer. At sufficiently high polyelectrolyte surface coverages, friction becomes insensitive to the level of chemical damage on the hair surface. Generally, the observed friction reductions were attributed to the break-up of SDS micelles initially adsorbed to CGG. The surfactants form a hydrophilic layer that leads to more water retention and reduced surface interdigitation, particularly at higher CGG adsorption densities. Most polyelectrolytes have flat, train-like conformations, particularly at high charge density and low surface coverage. The number of tail and loop conformations increases at lower charge density and higher surface coverage. However, this has a minor effect on friction compared to the amount of water and surfactant trapped inside the contact, thus suggesting that the formation of brushy looped polymer structures is not the main mechanism enabling friction reductions from polymer–surfactant deposition. The increased surface separation provided by higher CGG charge densities and particularly increased CGG adsorption density shifts the lubrication regime from boundary to mixed, leading to significantly lower friction.

We have shown how polyelectrolyte charge density and surface coverage affect adsorption, squeeze-out, and friction of polyelectrolyte–surfactant complexes on biomimetic hair surfaces. We employed a sequential adsorption approach, which should also be representative of polyelectrolyte–surfactant complexes adsorbed from aqueous solution.^19^ Systematic variation of the salt concentration and longer simulation timescales could help to investigate the role of counterion release in both sequentially adsorbed and pre-aggregated polymer–surfactant formulations – a mechanism found to be important in previous experiments^12,90^ but not observed in this study. Our methodology could also be adapted to virtually screen^91^ other sustainable polymers or surfactants, such as glycolipid biosurfactants,^18^ with respect to their hair conditioning and lubrication performance. More generally, by changing the surface model, the methodology can be readily extended to investigate other formulated products that contain polymer–surfactant complexes, such as fabric softeners^92^ and drug delivery agents.^93^

## Author contributions

E. W. methodology, investigation, data curation, formal analysis, visualization, writing – original draft; F. R.-R. conceptualization, project administration, writing – review & editing; S. A.-U. conceptualization, supervision, writing – review & editing; D. D. conceptualization, funding acquisition, resources, supervision, project administration, writing – review & editing; J. P. E. conceptualization, methodology, supervision, writing – original draft.

## Conflicts of interest

There are no conflicts of interest to declare.

## Supplementary Material

LF-003-D6LF00165C-s001

## Data Availability

The data supporting this article have been included as part of the supplementary information (SI). Supplementary information: mass density profiles at different CGG charge densities, CGG conformations in hair–hair contacts at different CGG charge densities, CGG adsorption snapshots at different CGG adsorption densities, guar swelling assessment on hair, film conformation changes at different simulation stages, overview table of relevant literature experiments compared to this work. See DOI: https://doi.org/10.1039/d6lf00165c.
