# Supplementary material for: Modifying polyelectrolyte charge density and surface coverage for enhanced polyelectrolyte–surfactant lubrication of biomimetic interfaces
Source: RSC Appl Interfaces. 2026 Jul 31;3(5):1588–605. doi: 10.1039/d6lf00165c (PMC13576499; doi:10.1039/d6lf00165c)
Supplement: Supplementary file 1 [file LF-003-D6LF00165C-s001.pdf]

Supplementary Information (SI) for RSC Applied Interfaces.
This journal is © The Royal Society of Chemistry 2026

Supporting Information for: Modifying polyelectrolyte charge density and surface coverage for enhanced polyelectrolyte-surfactant lubrication of biomimetic interfaces

Erik Weiand,[*,†,‡,¶] Francisco Rodriguez-Ropero,[§] Stefano Angioletti-Uberti,[‖,‡,¶] Daniele Dini,[†,‡,¶] and James P. Ewen[*,†,‡,¶]

†*Department of Mechanical Engineering, Imperial College London, South Kensington Campus, SW7 2AZ London, U.K.*

‡*Institute of Molecular Science and Engineering, Imperial College London, South Kensington Campus, SW7 2AZ London, U.K.*

¶*Thomas Young Centre for the Theory and Simulation of Materials, Imperial College London, South Kensington Campus, SW7 2AZ London, U.K.*

§*Corporate Functions Analytical and Data & Modeling Sciences, Mason Business Center, The Procter and Gamble Company, Mason, 45040 Ohio, U.S.A.*

‖*Department of Materials, Imperial College London, South Kensington Campus, SW7 2AZ London, U.K.*

E-mail: erik.weiand19@imperial.ac.uk; j.ewen@imperial.ac.uk

Mass density profiles at different CGG charge densities

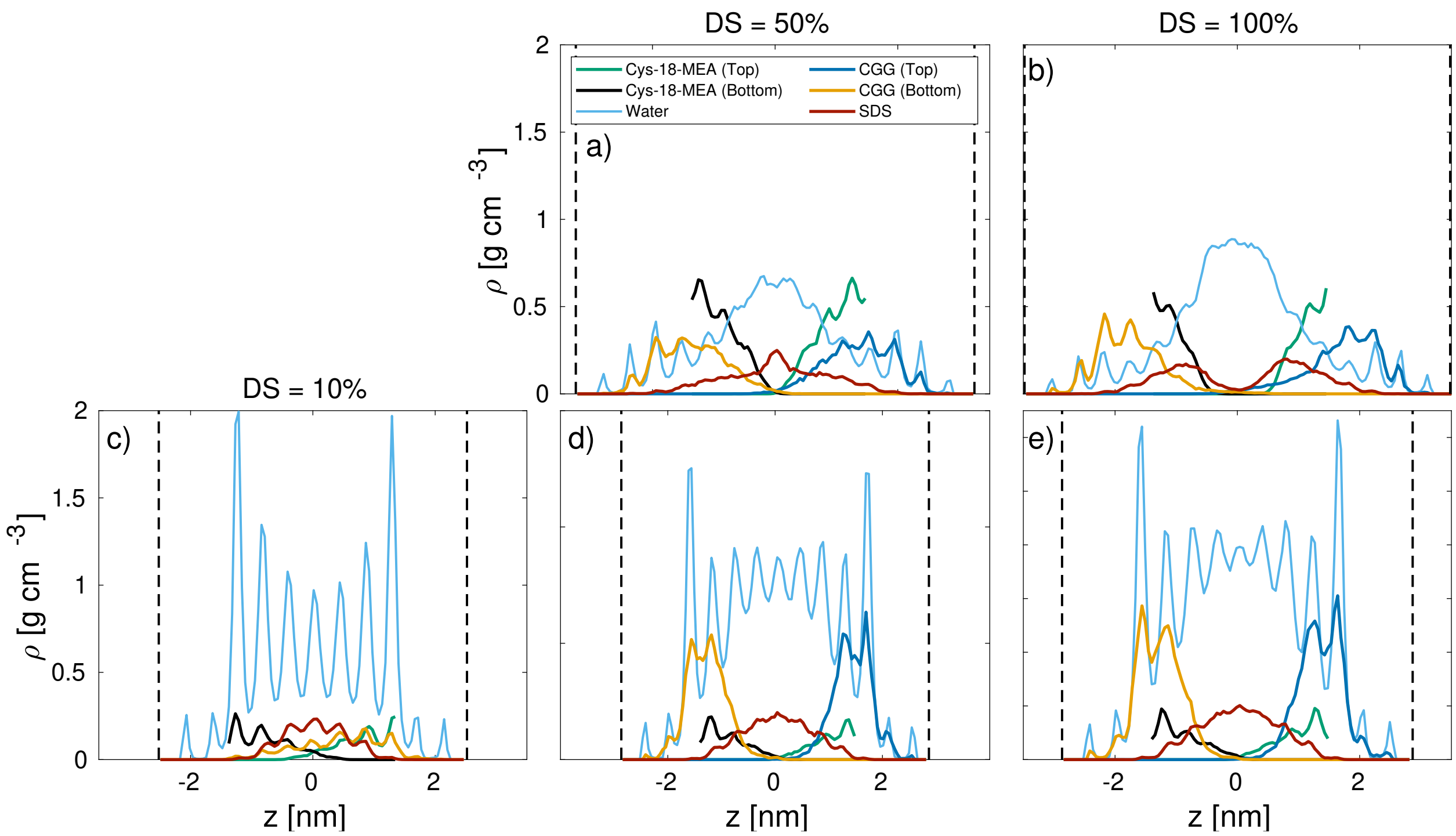

Figure S1: Mass densities profiles from NEMD at different charge densities (DS) on CGG with SDS on virgin (top row, a-b) and medium bleached hair (bottom row, c-e) at $\sigma = 10$ MPa and $v_s = 0.1$ m s^{-1}.

CGG conformations in hair-hair contacts at different CGG charge densities

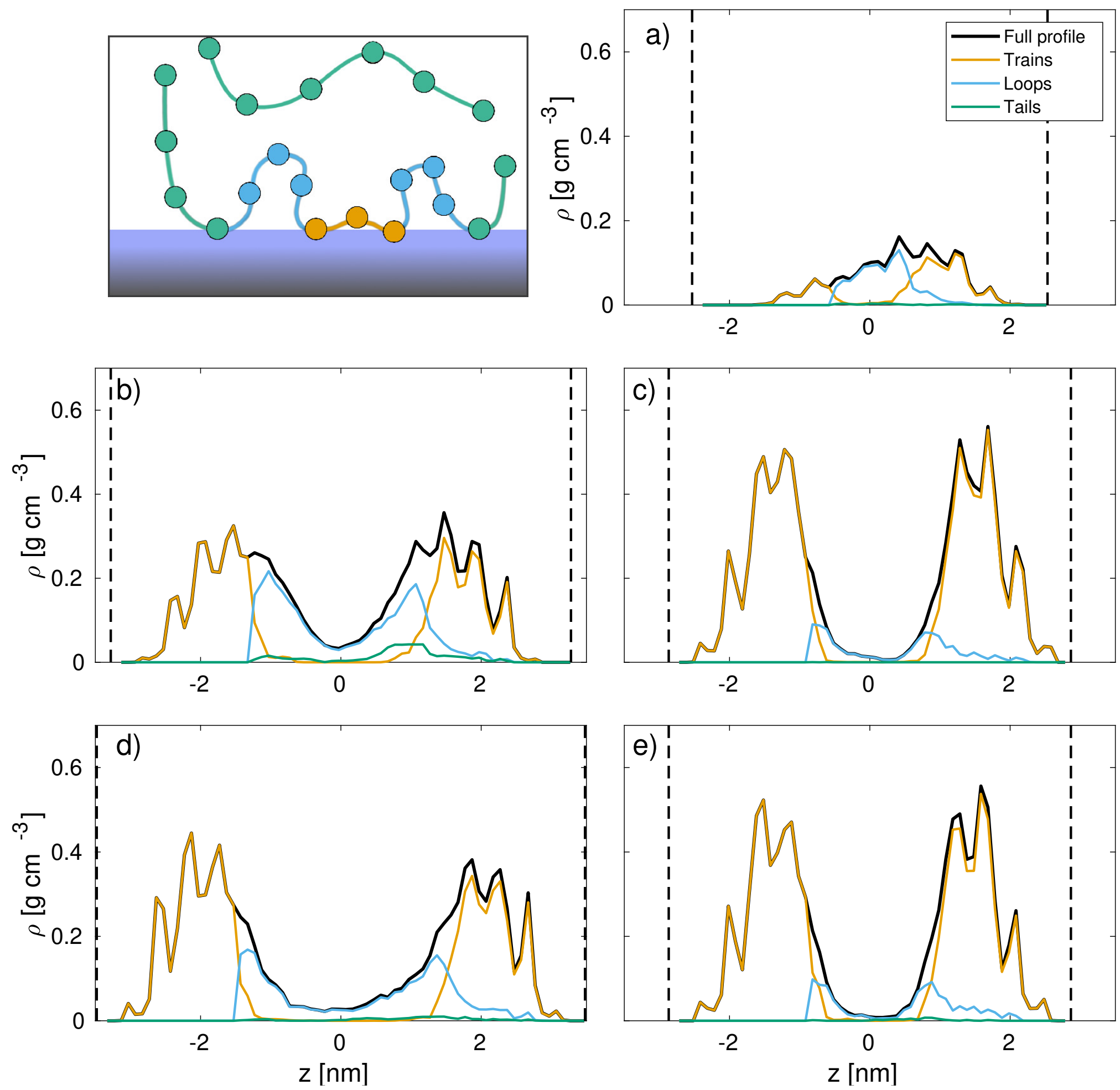

Figure S2: Time-averaged mass density profiles of CGG at different charge densities (DS), decomposed into trains, loops and tails during NEMD sliding simulations. Profiles are shown for virgin (left) and bleached hair (right) at (a) DS = 10%, (b-c) DS = 50% and (d-e) DS = 50%.

CGG adsorption at different CGG adsorption densities

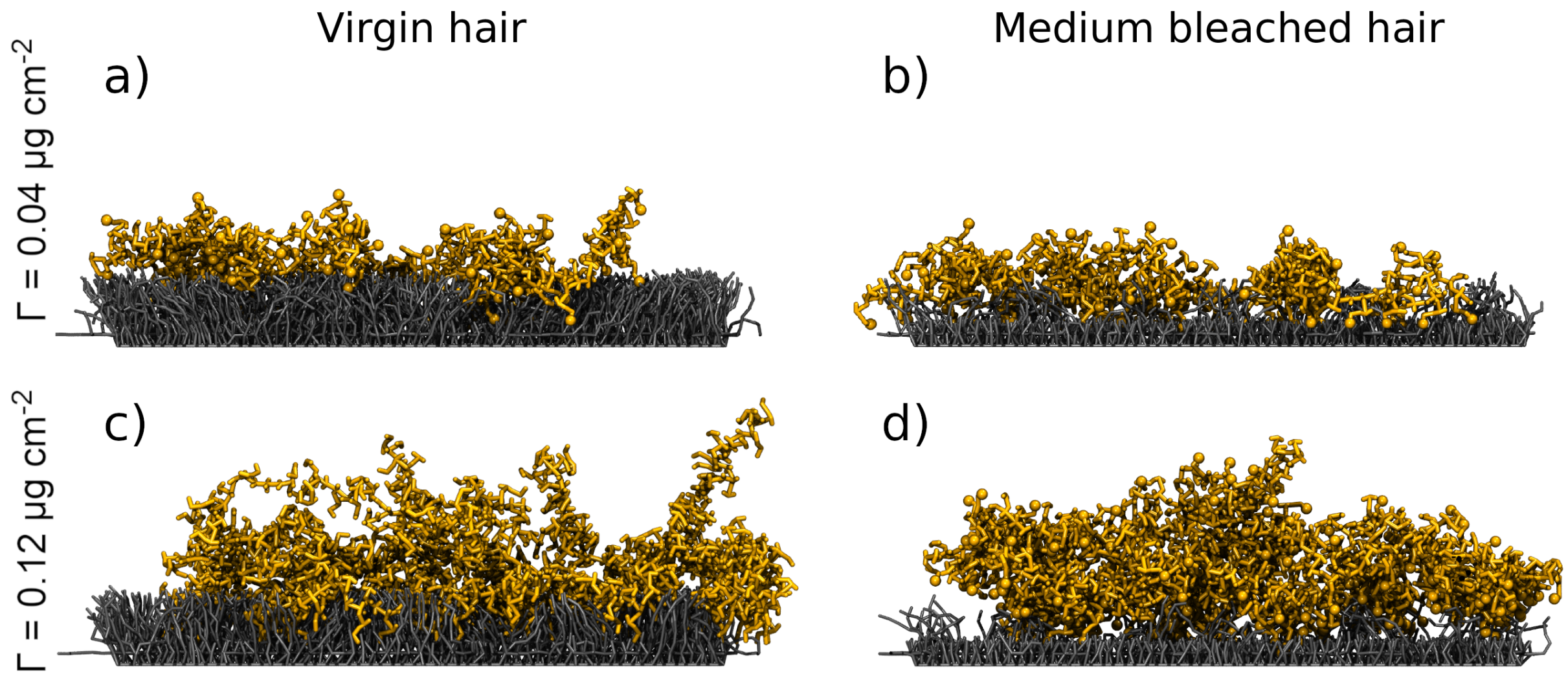

Figure S3: Snapshots of CGG (orange) at various adsorption densities, $\Gamma = 0.04$ μg cm^{-2} and $\Gamma = 0.12$ μg cm^{-2}, at the end of the CGG adsorption stage on virgin (left) and medium bleached hair (right). Rendered using VMD.[1]

Guar swelling on hair

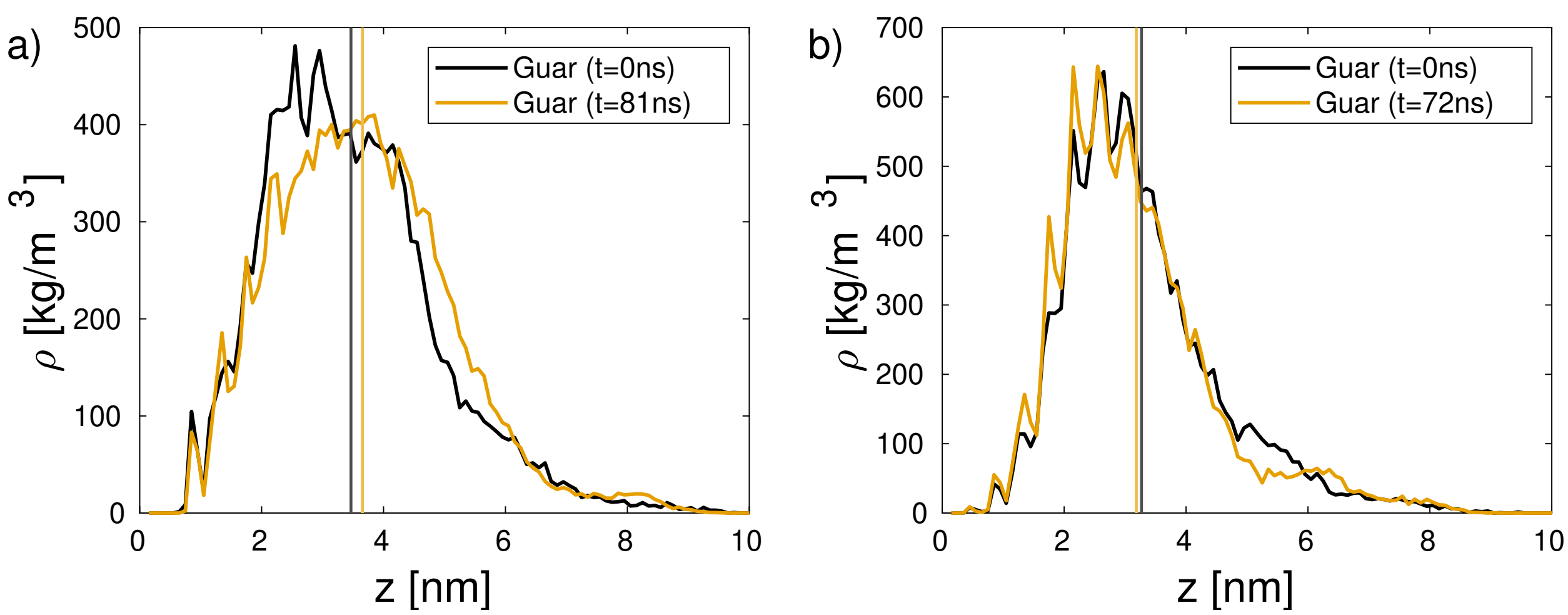

Figure S4: CGG mass density profile at $\Gamma = 0.12$ μg cm^{-2} on a) virgin and b) bleached hair in the presence of SDS as a function of time. Vertical lines indicate the guar center of mass at the respective time.

Film conformation changes at different simulation stages

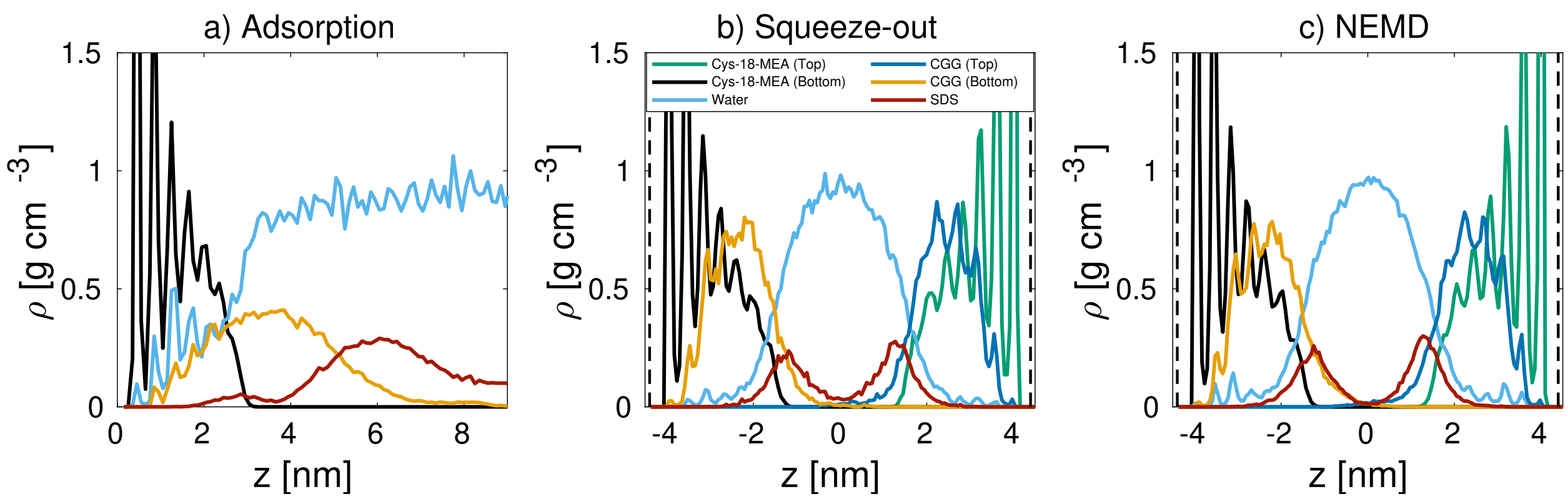

Figure S5: Time-averaged mass density profiles of CGG, SDS and the 18-MEA a) at the end of the adsorption stage, b) at the end of the squeeze-out stage, and c) during sliding for virgin hair surfaces at $\Gamma = 0.12$ μg cm^{-2}.

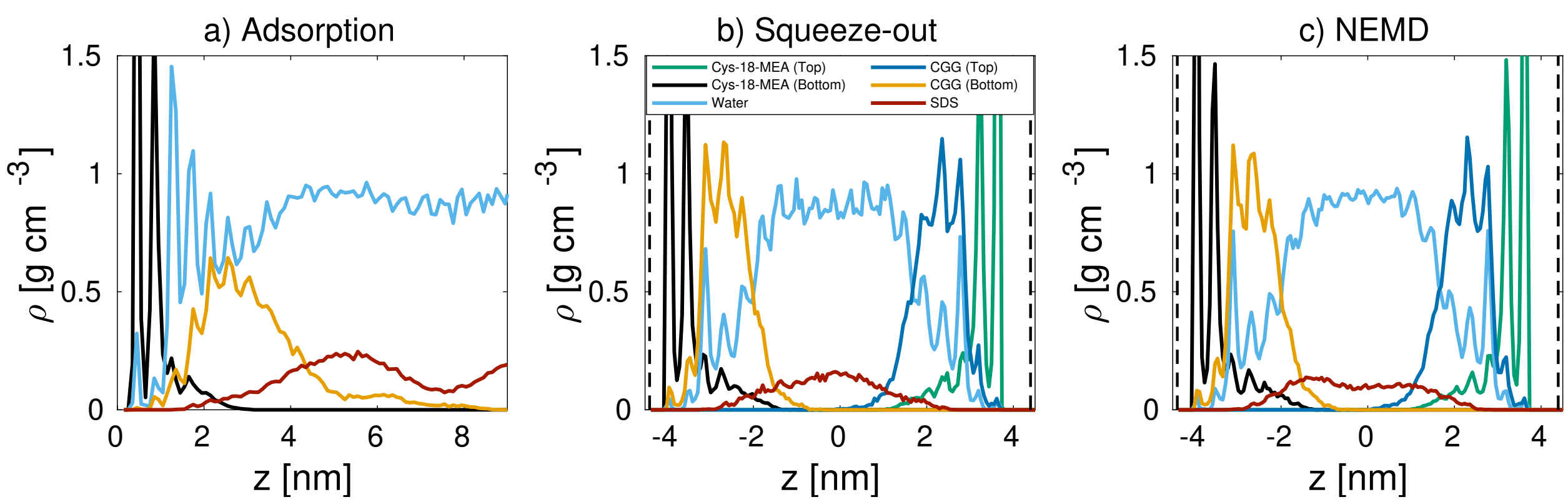

Figure S6: Time-averaged mass density profiles of CGG, SDS and the 18-MEA a) at the end of the adsorption stage, b) at the end of the squeeze-out stage, and c) during sliding for medium bleached hair surfaces at $\Gamma = 0.12$ μg cm^{-2}.

Table S1: Qualitative comparison between the CG-MD simulations in this work and literature experiments with high-charge-density polymers and oppositely-charged surfactants.

Reference	Polymer/ surfactant	Polymer M_w [kDa]	Polymer DS	Surface	Contact Thickness	Experiment type	Film thickness findings	Friction findings	COF
This work	CGG/SDS	36.9	10-100%	F-layer (hair)	4.3-8.8nm	MD	• Sequential adsorption<br>• Flat CGG conformations<br>• No CGG swelling with SDS<br>• CGG retains SDS	• Cooperative friction reductions<br>• Lubrication due to SDS & contact hydration	0.03-0.11
Hössel et al. (2000) [2]	CGG/SLES	n/a	n/a	Hair	n/a	Comb force	• Hypothesized loop formation<br>• Hypothesized over-conditioning with CGG	• Combing force reductions with CGG/ SLES addition	n/a
Dedinaite et al. (2000) [3]	PCMA/SDS	1,600	100%	Mica	n/a	SFA	• Slow convergence to reach equilibrium structures<br>• The order of adsorption can matter	n/a	n/a
Qian et al. (2004) [4]	Hydroxyethyl cellulose/SLES	400	66.6%	Mica	6-400nm	SFA	• Surfactant addition condenses polymer layer	• Surfactant addition increases friction due to polymer coiling	0.15-0.21
Dedinaite et al. (2010) [5]	PMAPTAC/SDS	480	100%	Mica	n/a	AFM	• Sequential adsorption<br>• Layer swelling with SDS addition<br>• Rod-like micelles wrapped by the polymer	• Friction reductions with SDS addition<br>• High layer fluidity required for lubricity	0.06-0.37

References

(1) Humphrey, W.; Dalke, A.; Schulten, K. VMD: Visual molecular dynamics. *Journal of Molecular Graphics and Modelling* **1996**, *14*, 33–38.

(2) Hössel, P.; Dieing, R.; Nörenberg, R.; Pfau, A.; Sander, R. Conditioning polymers in today's shampoo formulations - Efficacy, mechanism and test methods. *International Journal of Cosmetic Science* **2000**, *22*, 1–10.

(3) Dedinaite, A.; Claesson, P. M.; Bergström, M. Polyelectrolyte-surfactant layers: adsorption of preformed aggregates versus adsorption of surfactant to preadsorbed polyelectrolyte. *Langmuir* **2000**, *16*, 5257–5266.

(4) Qian, L.; Charlot, M.; Perez, E.; Luengo, G.; Potter, A.; Cazeneuve, C. Dynamic Friction by Polymer/Surfactant Mixtures Adsorbed on Surfaces. *Journal of Physical Chemistry B* **2004**, *108*, 18608–18614.

(5) Dedinaite, A.; Pettersson, T.; Mohanty, B.; Claesson, P. M. Lubrication by organized soft matter. *Soft Matter* **2010**, *6*, 1520–1526.
